# Supplementary material for: Comparative mucomic analysis of three functionally distinct Cornu aspersum Secretions
Source: Nat Commun. 2023 Sep 2;14:5361. doi: 10.1038/s41467-023-41094-z (PMC10475054; doi:10.1038/s41467-023-41094-z)
Supplement: Supplementary file 1 — Supplementary Information [file 41467_2023_41094_MOESM1_ESM.pdf]

Supplementary Information

Comparative Mucomic Analysis of Three Functionally Distinct *Cornu aspersum* Secretions

Antonio R. Cerullo,[1,2,3] Maxwell B. McDermott,[3†] Lauren E. Pepi,[4†] Zhi-Lun Liu,[1,5] Diariou Barry,[1] Sheng Zhang,[1] Xu Yang,[4] Xi Chen,[1,5,6,7] Parastoo Azadi,[4] Mande Holford,[2,3,6,8,9] Adam B. Braunschweig*[1,2,3,6]

[1] The Advanced Science Research Center, Graduate Center of the City University of New York, 85 St. Nicholas Terrace, New York, New York 10031, USA
[2] The PhD Program in Biochemistry, Graduate Center of the City University of New York, 365 Fifth Avenue, New York, New York 10016, USA
[3] Department of Chemistry and Biochemistry, Hunter College, 695 Park Avenue, New York, New York 10065, USA
[4] Complex Carbohydrate Research Center, University of Georgia, 315 Riverbend Road, Athens, Georgia 30602, USA.
[5] Department of Chemical Engineering, The City College of New York, New York, New York 10031, USA
[6] The PhD Program in Chemistry, Graduate Center of the City University of New York, 365 Fifth Avenue, New York, New York 10016, USA
[7] The PhD Program in Physics, Graduate Center of the City University of New York, 365 Fifth Avenue, New York, New York 10016, USA
[8] The PhD Program in Biology, Graduate Center of the City University of New York, 365 Fifth Avenue, New York, New York 10016, USA
[9] Department of Invertebrate Zoology, The American Museum of Natural History, New York, New York 10024, USA
[†] indicates authors contributed equally to this work

Email: abraunschweig@gc.cuny.edu

Table of Contents

1. Supplementary Figures……………………………..…………………… ….….…….S3
2. Supplementary Tables…………………………………………………………..S38
3. Supplementary Methods………… ………………………………………………….S51
4. Supplementary References …...……………………………………...……..………..S62

Supplementary Figures

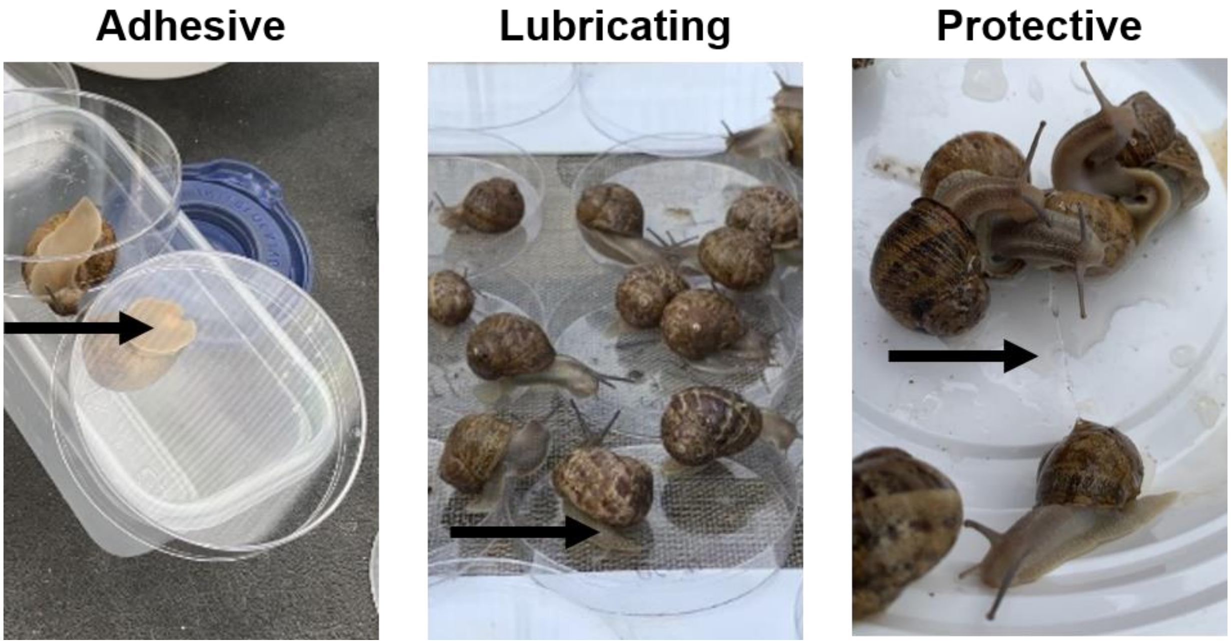

Supplementary Figure 1. Collection of mucus from *C. aspersum* snails. Arrows point to secreted mucus.

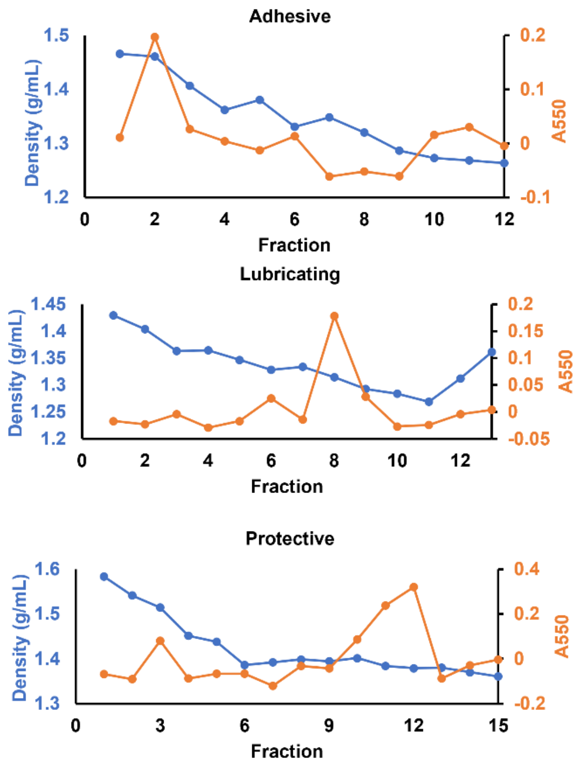

Supplementary Figure 2. Fractionation of snail mucus proteins through isopycnic density gradient ultracentrifugation. Blue curves represent measured density of each fraction. Orange curves represent periodic acid-Schiff's stain (PAS) response using a previously established microtiter plate assay.[5]

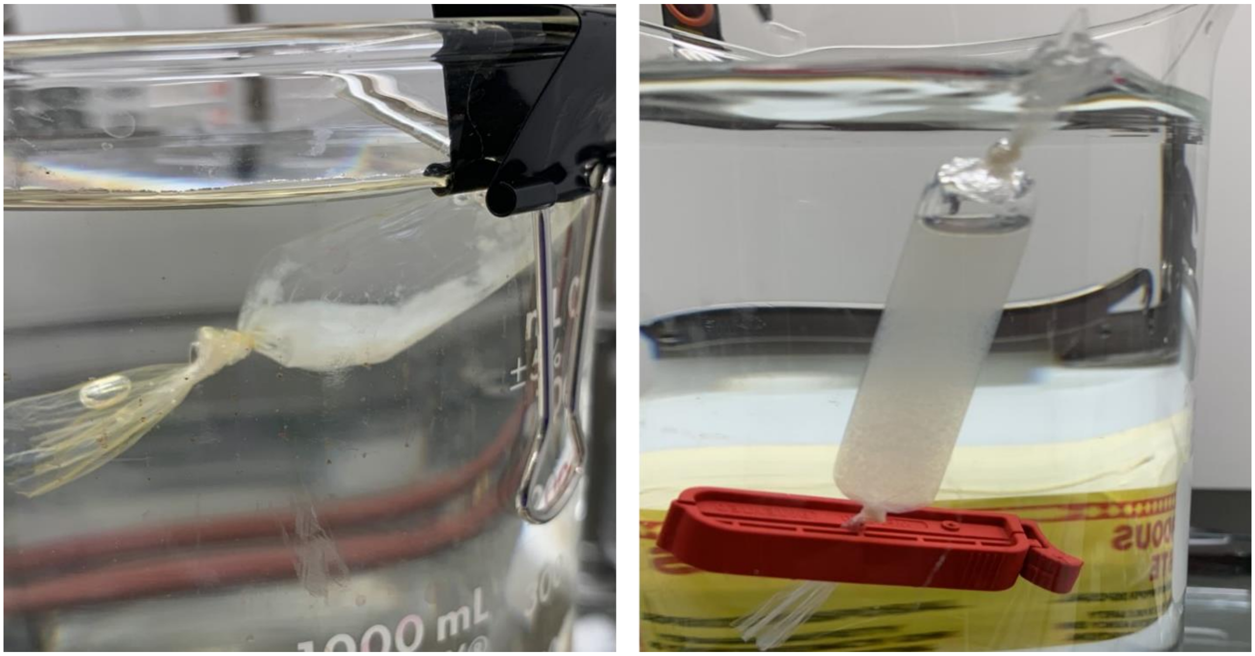

Supplementary Figure 3. Mucus samples post-dialysis. Flocculent beige precipitate forms after dialyzing mucus against ultrapure water from Guandinium hydrochloride solution. Left image is after allowing sample to sit undisturbed for at least 30 min so that precipitate sediments. Right image is immediately after mixing, dispersing protein evenly throughout the suspension. Solutions were clear and colorless prior to dialysis.

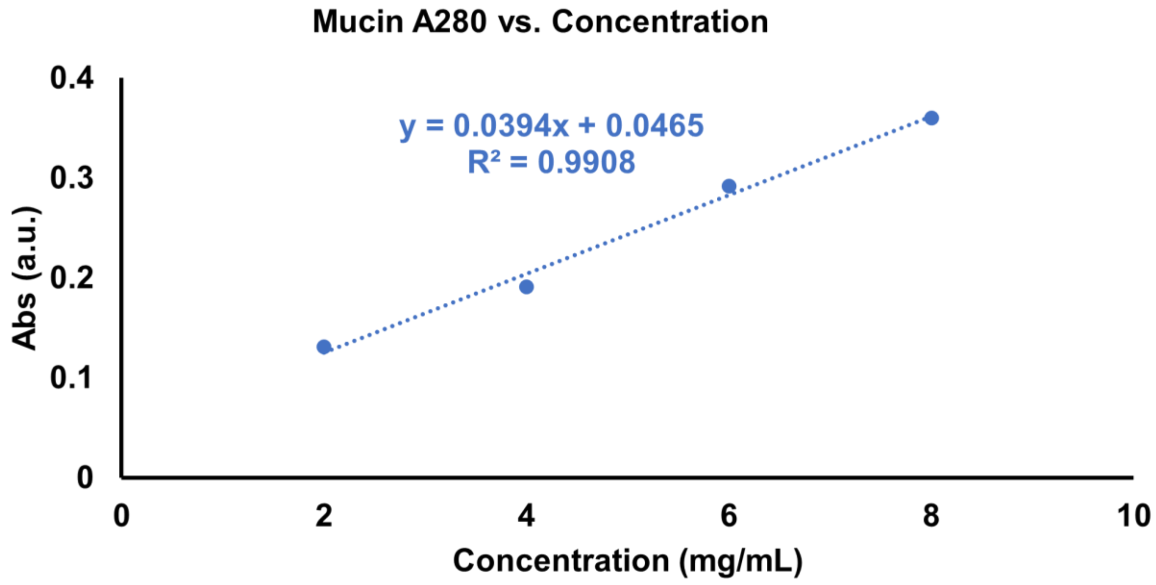

Supplementary Figure 4. Mucus standard curve for both purified mucus proteins determined by Nanodrop spectrophotometric analysis using a linear fit.

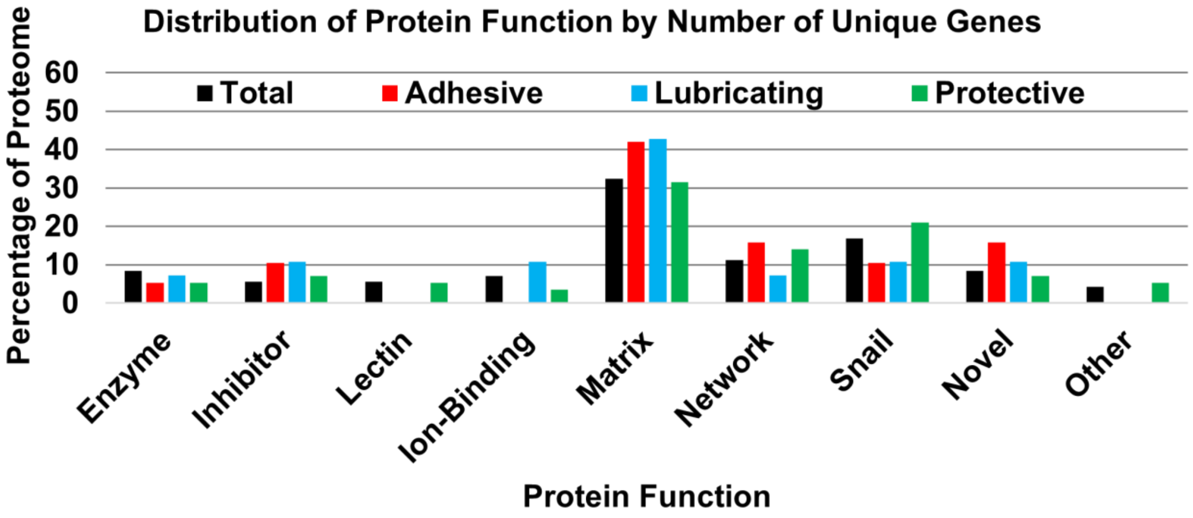

Supplementary Figure 5. Percentage of unique genes for protein function found. Percentages were calculated by finding the ratio of the number of unique genes for each function to the total number of genes within each mucus sample. "Snail" refers to proteins without any determinable function but had structural similarity to uncharacterized proteins previously found in snails. "Novel" indicates the protein had no similarity to any known proteins in the NCBI nor PFAM databases.

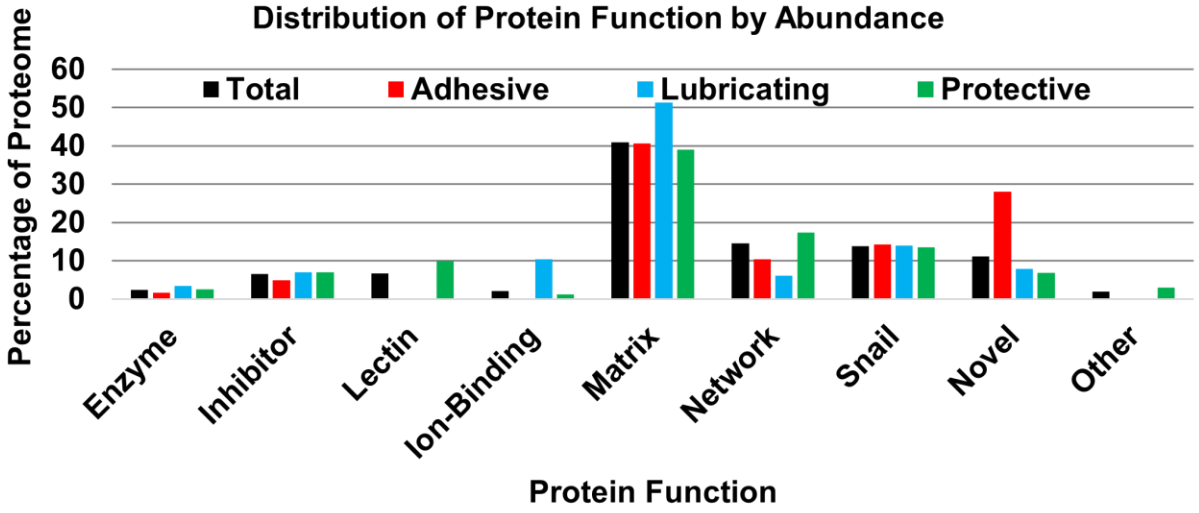

Supplementary Figure 6. Percentage of protein abundance for each function found. Percentages were calculated by finding the ratio of the number of MS/MS counts for all genes within each function to the total number of MS/MS counts within each mucus sample. “Snail” refers to proteins without any determinable function, but had structural similarity to uncharacterized proteins previously found in snails. “Novel” indicates the protein had no similarity to any known proteins in the NCBI nor PFAM databases.

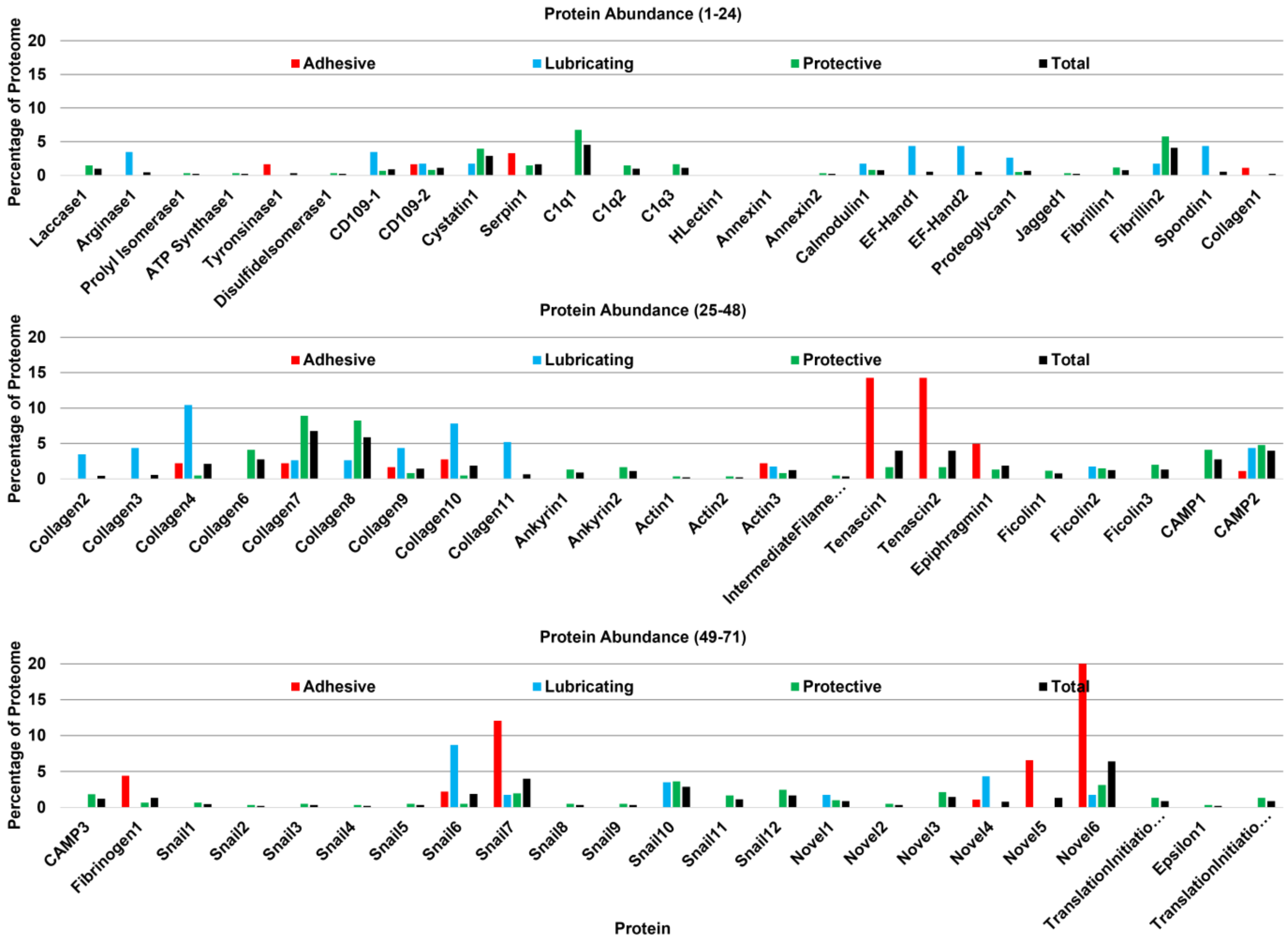

Supplementary Figure 7. Abundances for all 71 proteins identified. Percentages were calculated by finding the ratio of the number of MS/MS counts for each gene to the total number of MS/MS counts within each mucus sample. “Snail” refers to proteins without any determinable function but had structural similarity to uncharacterized proteins previously found in snails. “Novel” indicates the protein had no similarity to any known proteins in the NCBI nor PFAM databases.

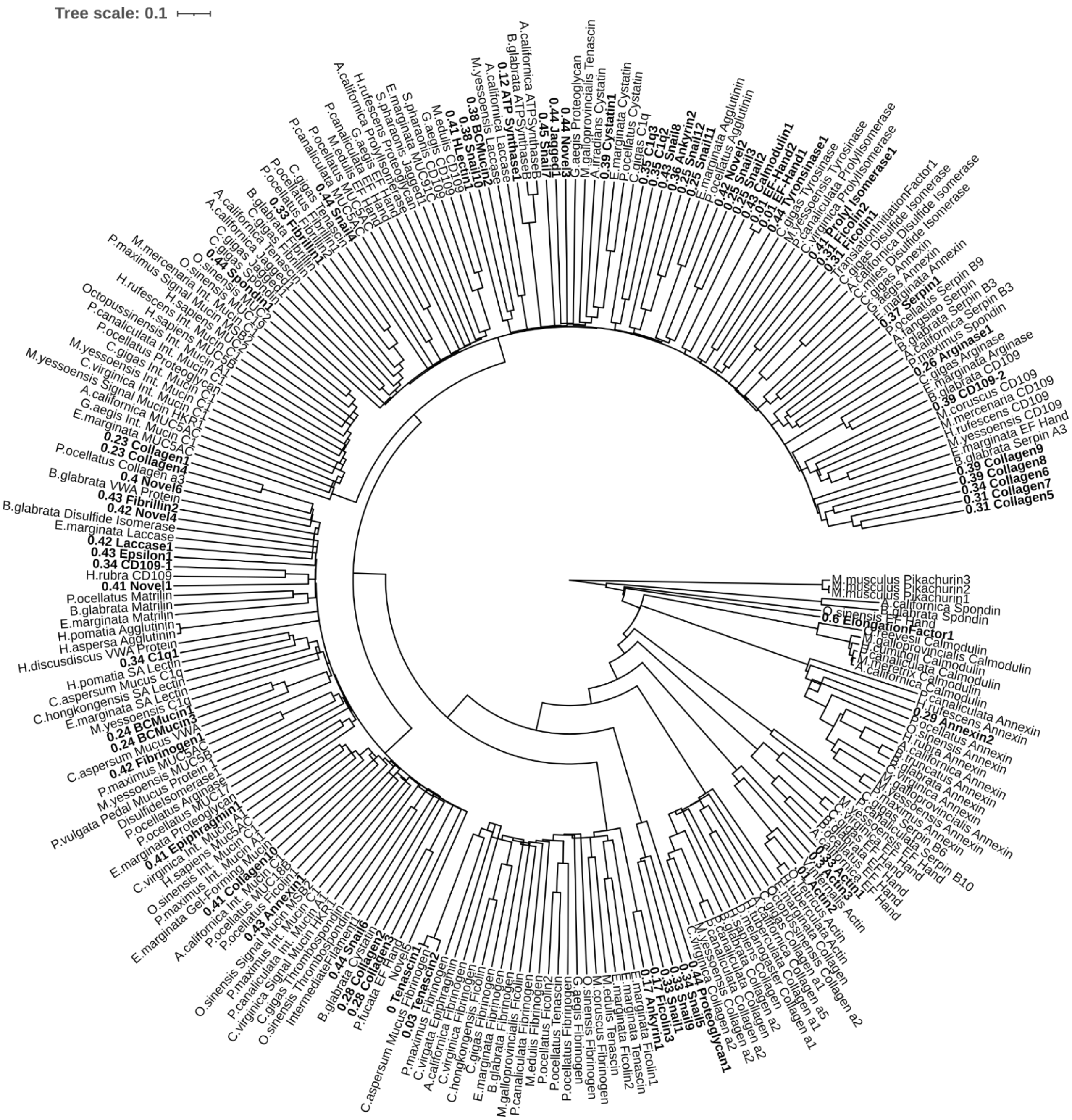

Supplementary Figure 8. Dendrogram of identified snail mucus proteins based on sequence similarity. Topology is identical to the dendrogram shown in Figure 2. The 71 proteins identified in this study are bolded with branch lengths shown. Protein sequences from *Amphioctopus fangsiao, Aplysia californica, Argopecten irradians, Biomphalaria glabrata, Bulinus truncates, Cernuella virgata, Cornu aspersum, Crassostrea gigas, Crassostrea hongkongensis, Crassostrea virginica, Elysia marginata, Gigantopelta aegis, Haliotis discus, Haliotis rubra, Haliotis tuberculata, Helix pomatia, Hemitoma cumingii, Mercenaria mercenaria, Meretrix meretrix, Mizuhopecten yessoensis, Mus musculus, Mytilus coruscus, Mytilus edulis, Mytilus galloprovincialis, Octopus sinensis, Onchidium reevesii, Patella vulgata, Pectin Maximus, Pinctada fucata, Plakobranchus ocellatus, Pomacea canaliculata, Sepia pharaonis,* and *Vampyroteuthis infernalis*, were used to generate the dendrogram.

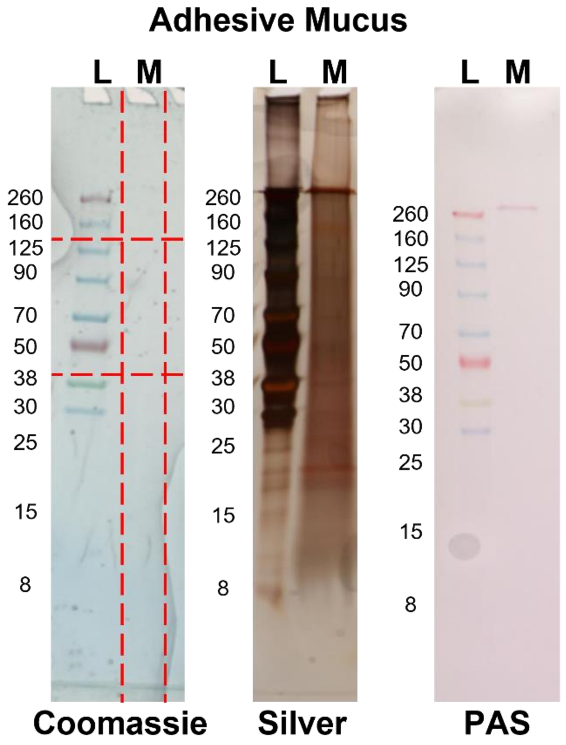

Supplementary Figure 9. SDS-PAGE of adhesive snail mucus using a 15-well 4 – 20 % Tris-glycine precast gradient gel (BioRad, 4561096). Samples (M lanes) were run in triplicate alongside a Chameleon Duo Pre-Stained protein ladder (LI-COR, 928-60000; L lane) and separately stained with Coomassie (left), Silver (middle), and PAS (right). Coomassie-stained M lanes containing purified mucus proteins were sliced into low (< 40 kDa), medium (40 – 150 kDa), and high (150+ kDa) slices, as indicated by red dashed lines, and these slices were used for proteomic analysis. Source data are provided as a Source Data file.

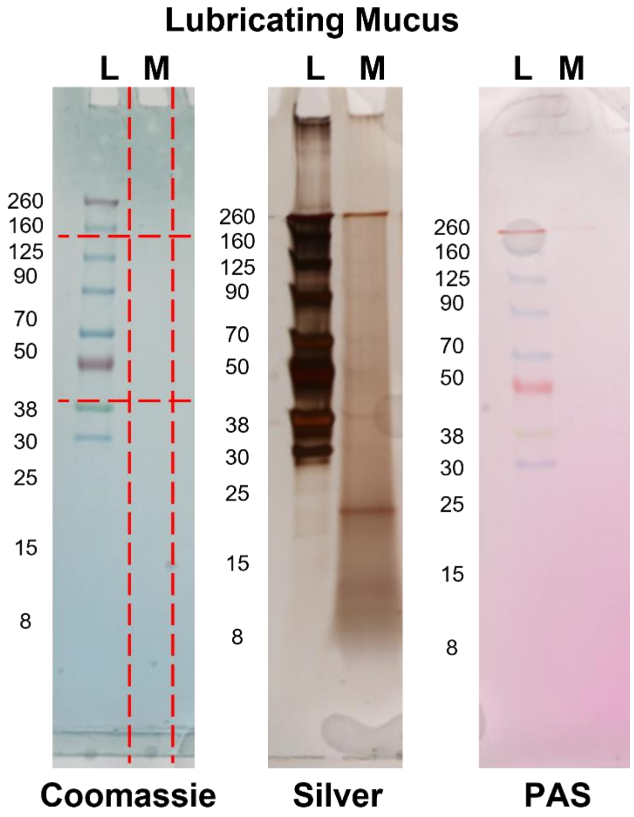

Supplementary Figure 10. SDS-PAGE of lubricating snail mucus using a 15-well 4 – 20 % Tris-glycine precast gradient gel (BioRad, 4561096). Samples (M lanes) were run in triplicate alongside a Chameleon Duo Pre-Stained protein ladder (LI-COR, 928-60000; L lane) and separately stained with Coomassie (left), Silver (middle), and PAS (right). Coomassie-stained M lanes containing purified mucus proteins were sliced into low (< 40 kDa), medium (40 – 150 kDa), and high (150+ kDa) slices, as indicated by red dashed lines, and these slices were used for proteomic analysis. Source data are provided as a Source Data file.

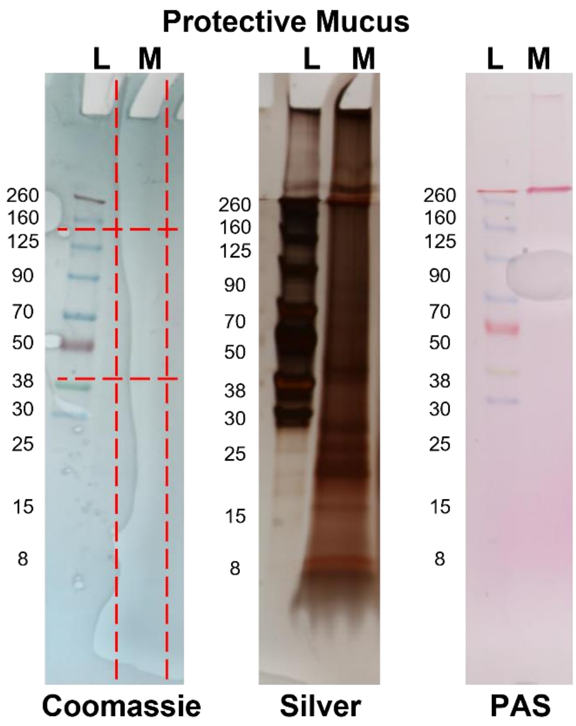

Supplementary Figure 11. SDS-PAGE of protective snail mucus using a 15-well 4 – 20 % Tris-glycine precast gradient gel (BioRad, 4561096). Samples (M lanes) were run in triplicate alongside a Chameleon Duo Pre-Stained protein ladder (LI-COR, 928-60000; L lane) and separately stained with Coomassie (left), Silver (middle), and PAS (right). Coomassie-stained M lanes containing purified mucus proteins were sliced into low (< 40 kDa), medium (40 – 150 kDa), and high (150+ kDa) slices, as indicated by red dashed lines, and these slices were used for proteomic analysis. Source data are provided as a Source Data file.

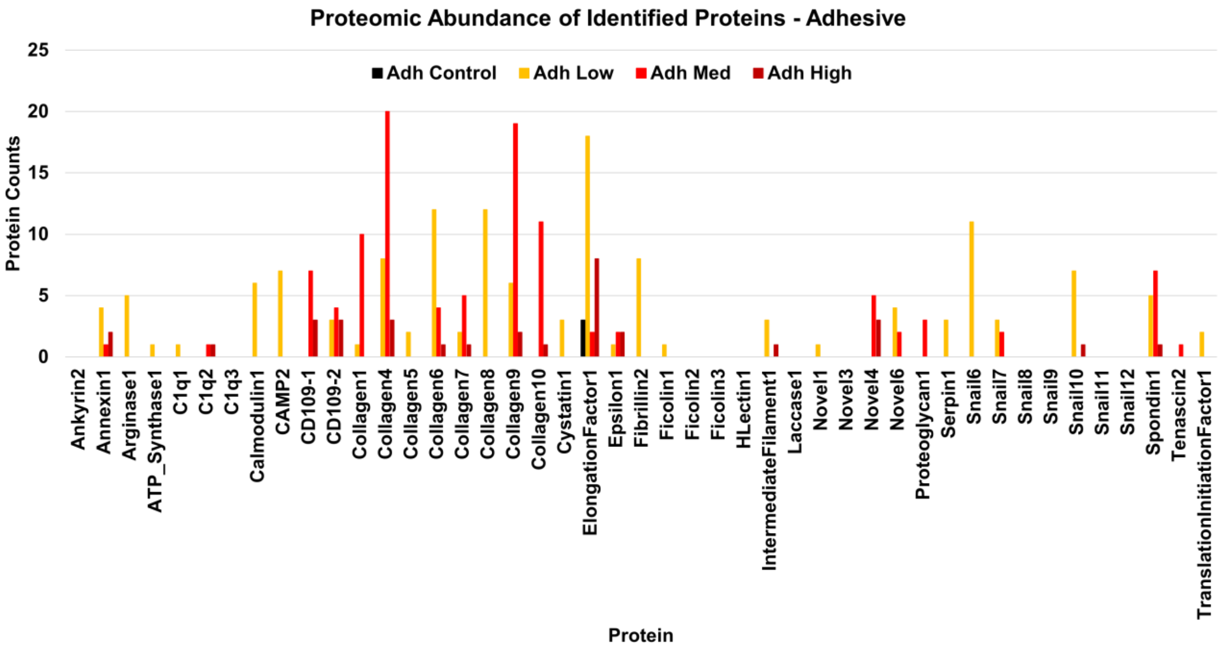

Supplementary Figure 12. Proteomic abundance of SDS-PAGE-resolved adhesive (Adh) mucus proteins. Control refers to a gel slice that did not contain protein. Low, Med, and High refers to gel slices that contained proteins of molecular mass < 40 kDa, 40 – 150 kDa, and 150+ kDa, respectively.

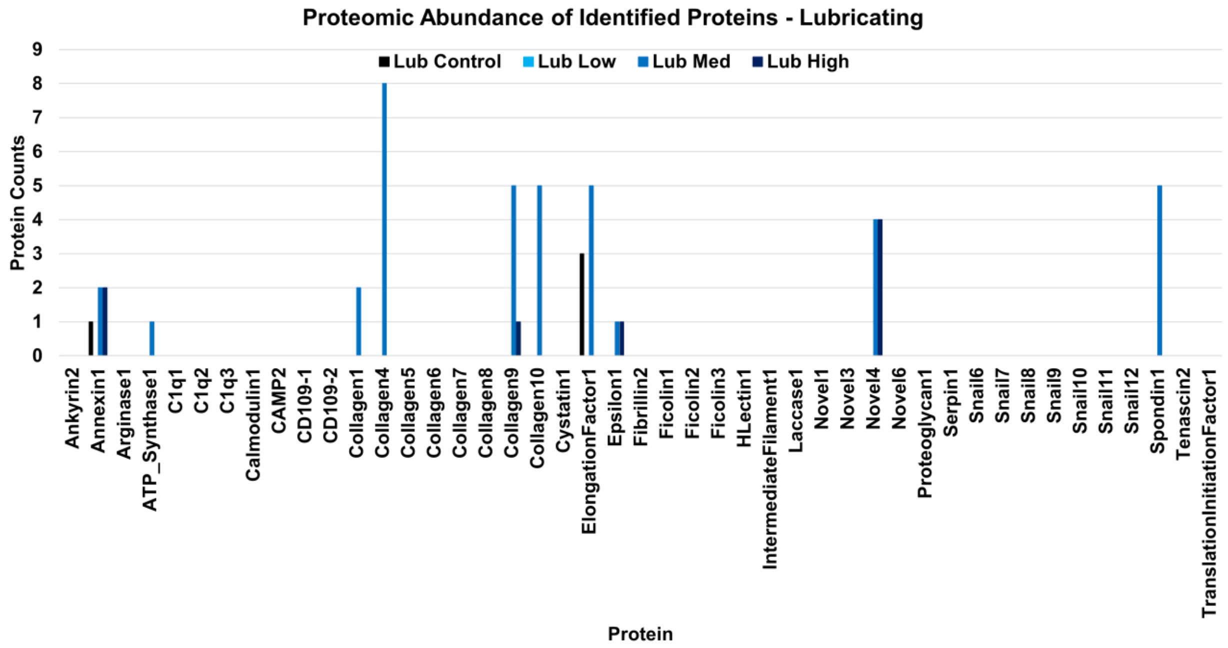

Supplementary Figure 13. Proteomic abundance of SDS-PAGE-resolved lubricating (Lub) mucus proteins. Control refers to a gel slice that did not contain protein. Low, Med, and High refers to to gel slices that contained proteins of molecular mass molecular mass < 40 kDa, 40 – 150 kDa, and 150+ kDa, respectively.

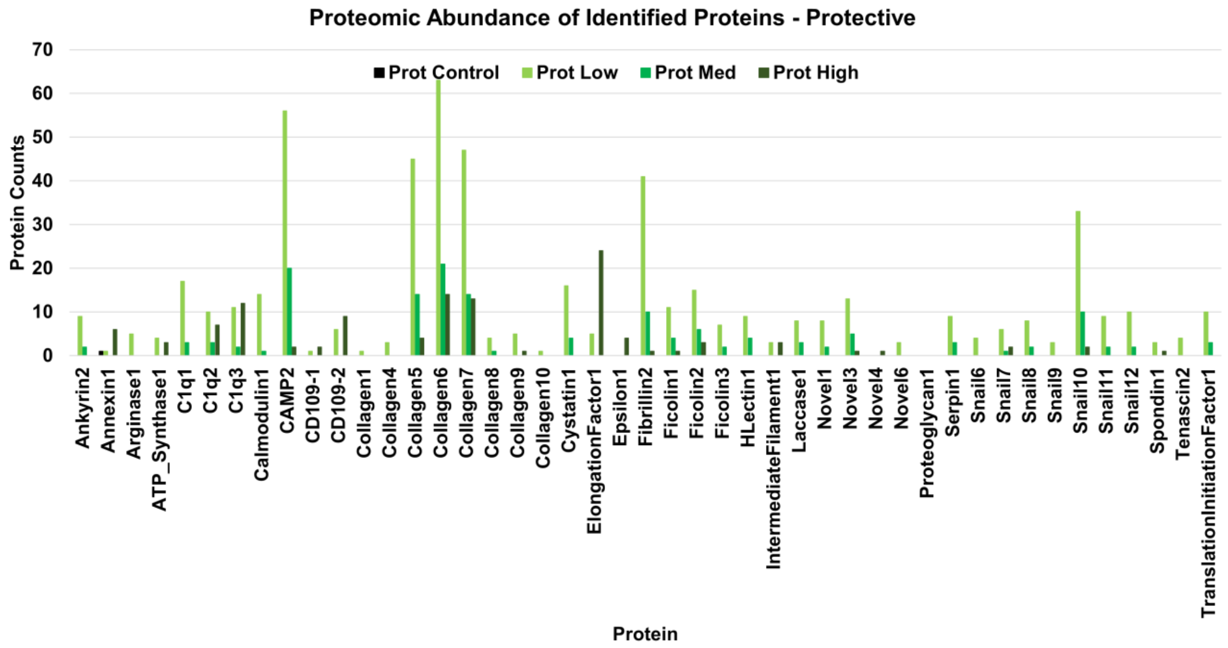

Supplementary Figure 14. Proteomic abundance of SDS-PAGE-resolved protective (Prot) mucus proteins. Control refers to a gel slice that did not contain protein. Low, Med, and High refers to to gel slices that contained proteins of molecular mass molecular mass < 40 kDa, 40 – 150 kDa, and 150+ kDa, respectively.

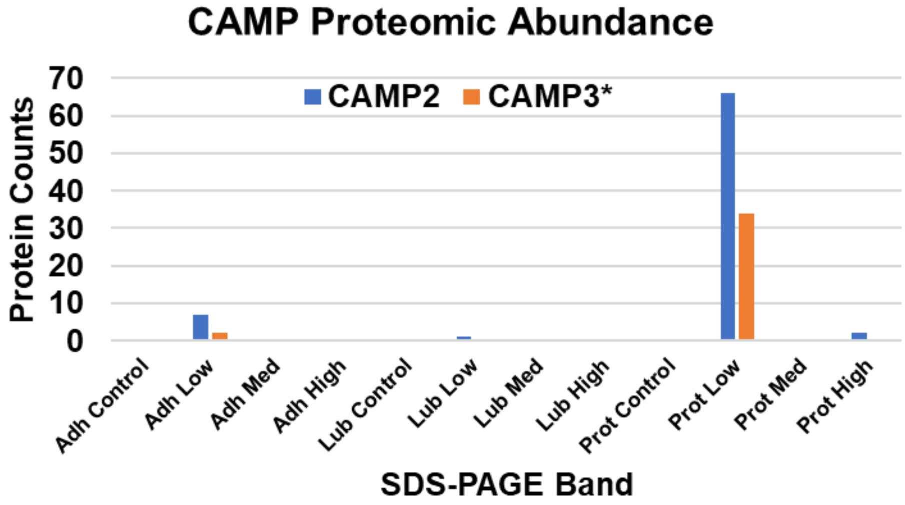

Supplementary Figure 15. Proteomic abundance of CAMP2 and CAMP3 across the three purified snail mucus. Control refers to a gel slice that did not contain protein. Low, Med, and High refers to molecular mass of < 40 kDa, 40 – 150 kDa, and 150+ kDa, respectively. Adh: adhesive snail mucus; Lub: lubricating snail mucus; Prot: protective snail mucus.

*An isoform of CAMP3 was detected in the SDS-PAGE analysis rather than the original CAMP3 gene identified in the proteomics analysis, which does not appear in Supplementary Figures 12 – 14.

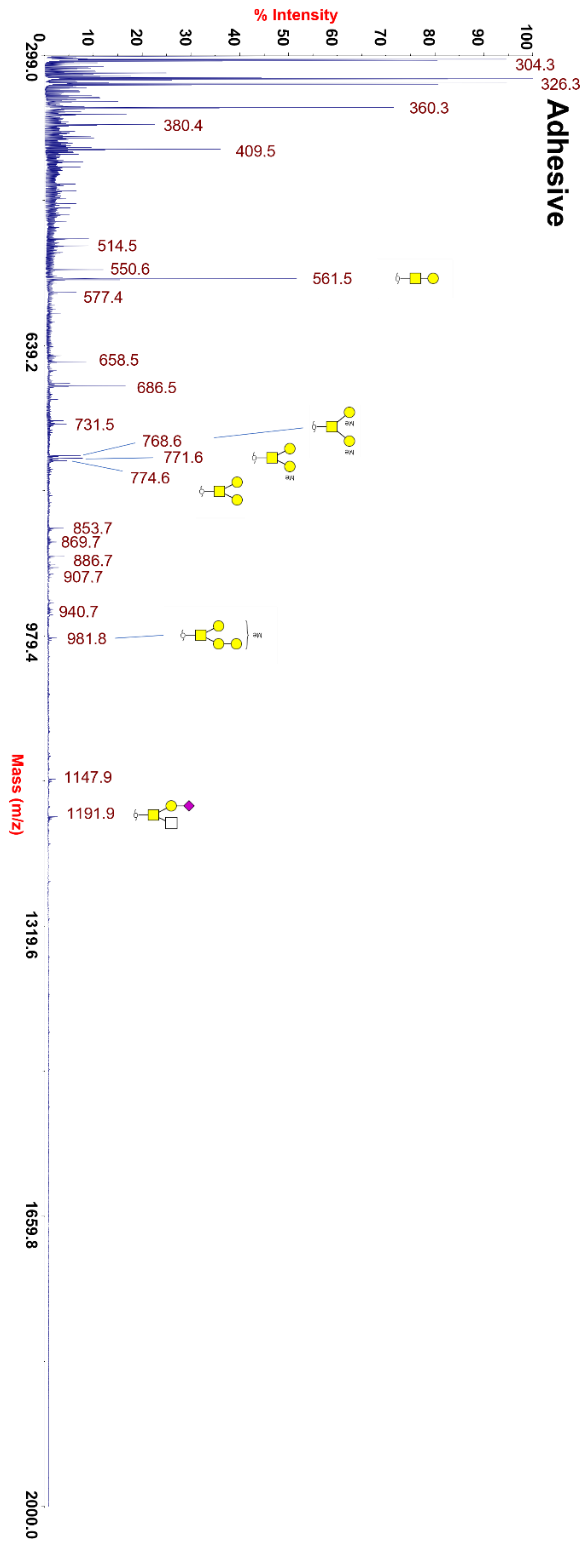

Supplementary Figure 16. Glycomic mass spectrum showing m/z peaks and compositional assignments of extracted *O*-glycans from *C. aspersum* adhesive mucus samples. Samples were per-deuter-*O*-methylated and adducts shown are Na+.

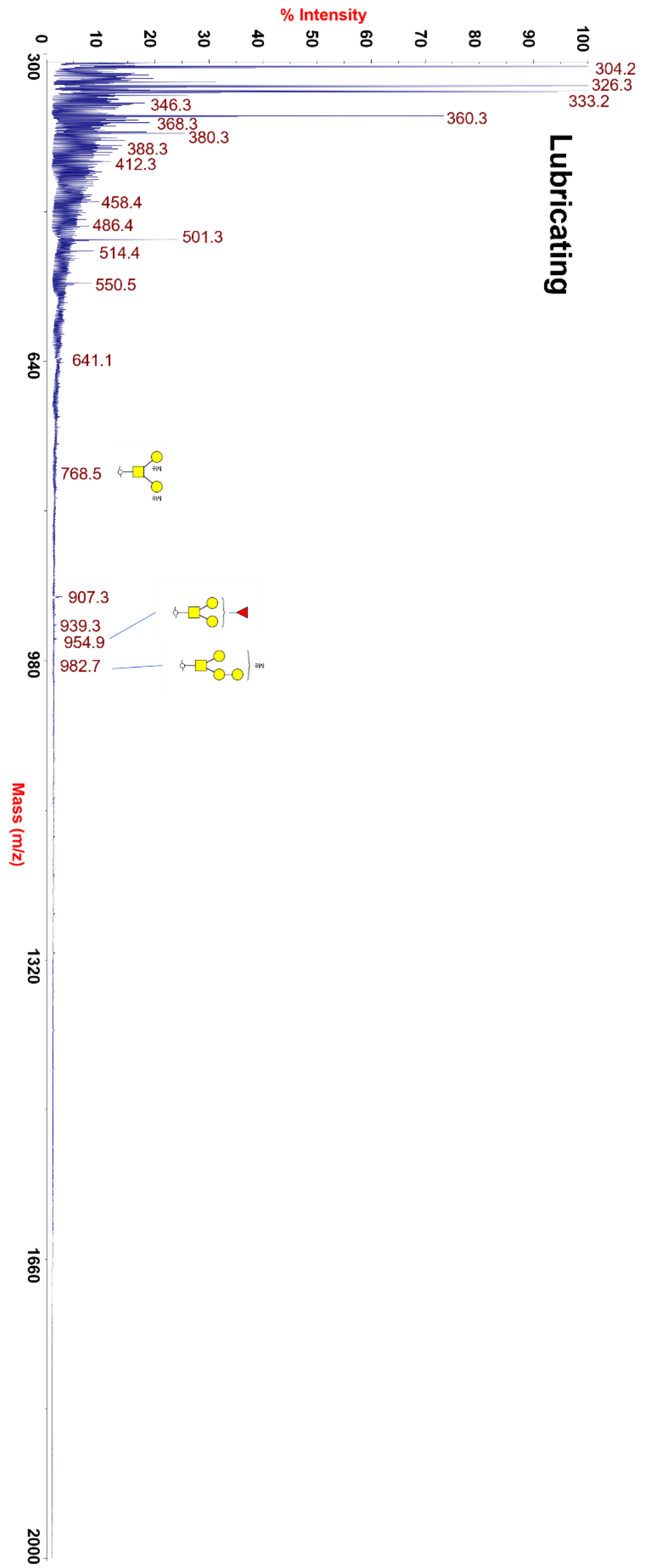

Supplementary Figure 17. Glycomic mass spectrum showing m/z peaks and compositional assignments of extracted *O*-glycans from *C. aspersum* lubricating mucus samples. Samples were per-deuter-*O*-methylated and adducts shown are Na+.

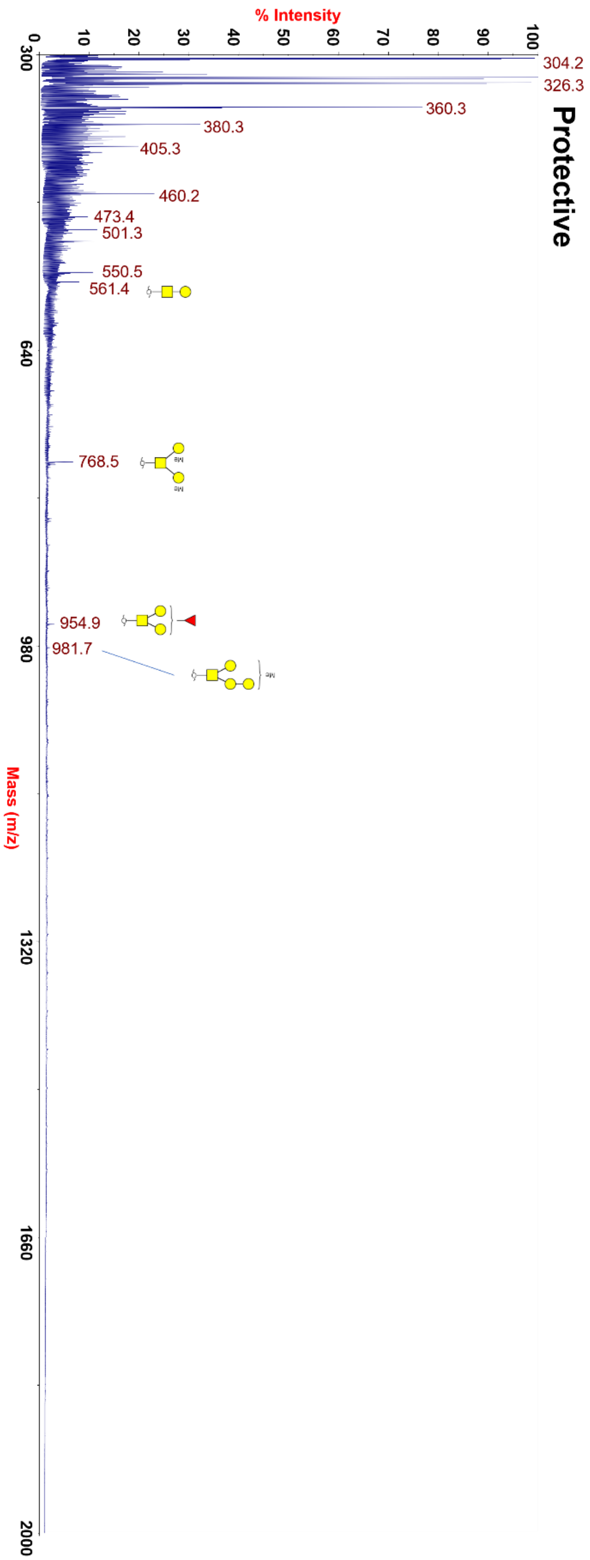

Supplementary Figure 18. Glycomic mass spectrum showing m/z peaks and compositional assignments of extracted *O*-glycans from *C. aspersum* protective mucus samples. Samples were per-deuter-*O*-methylated and adducts shown are Na+.

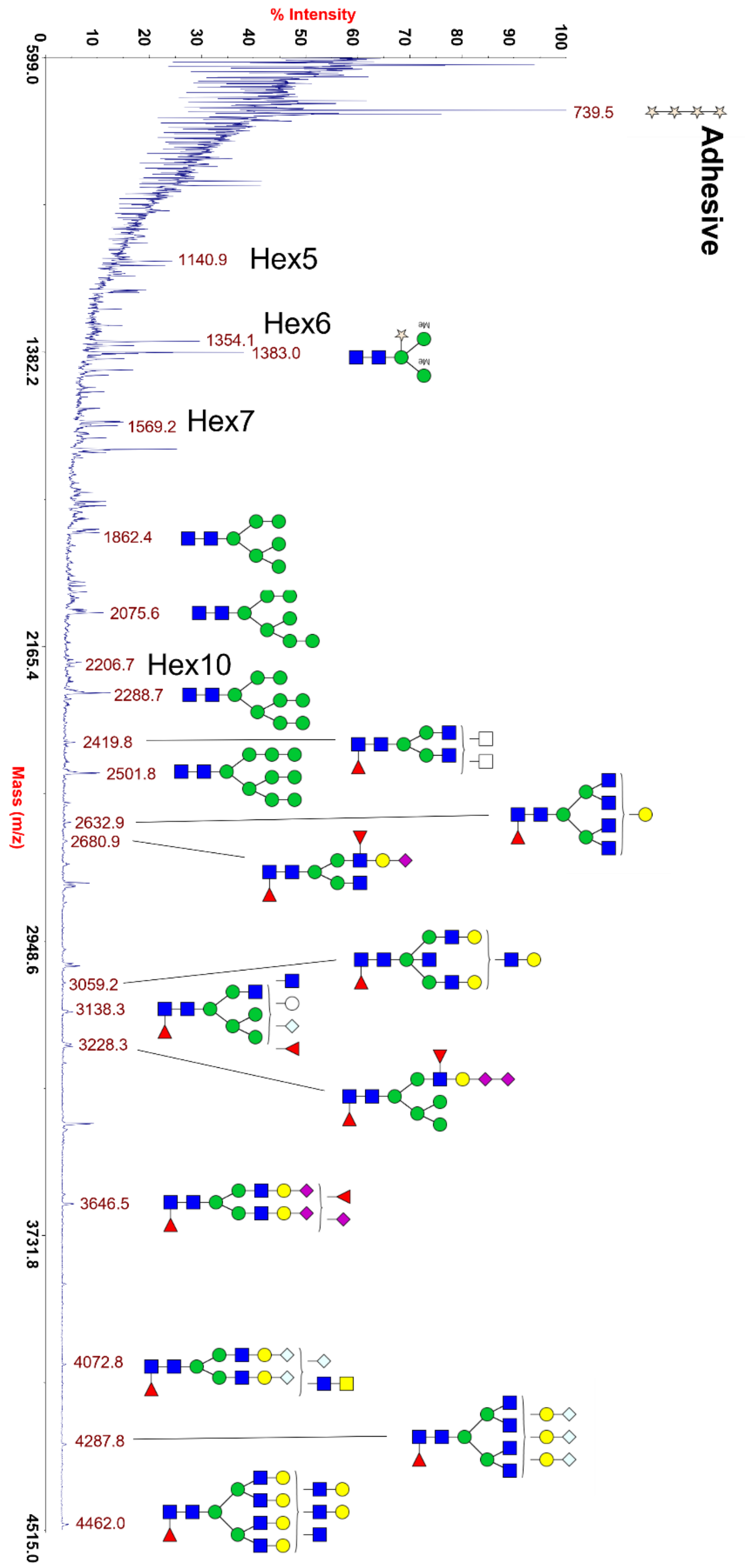

Supplementary Figure 19. Glycomic mass spectrum showing m/z peaks and compositional assignments of extracted *N*-glycans from *C. aspersum* adhesive mucus samples. Samples were per-deuter-*O*-methylated and adducts shown are Na+.

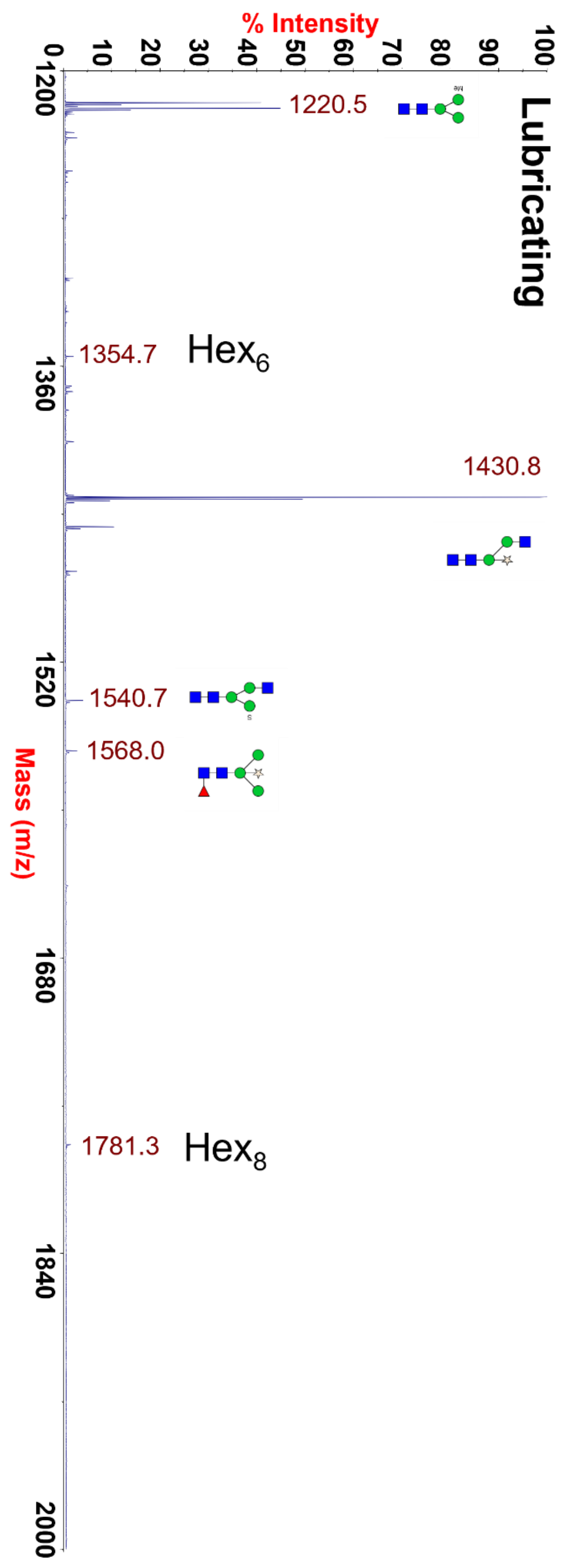

Supplementary Figure 20. Glycomic mass spectrum showing m/z peaks and compositional assignments of extracted *N*-glycans from *C. aspersum* lubricating mucus samples. Samples were per-deuter-*O*-methylated and adducts shown are Na+.

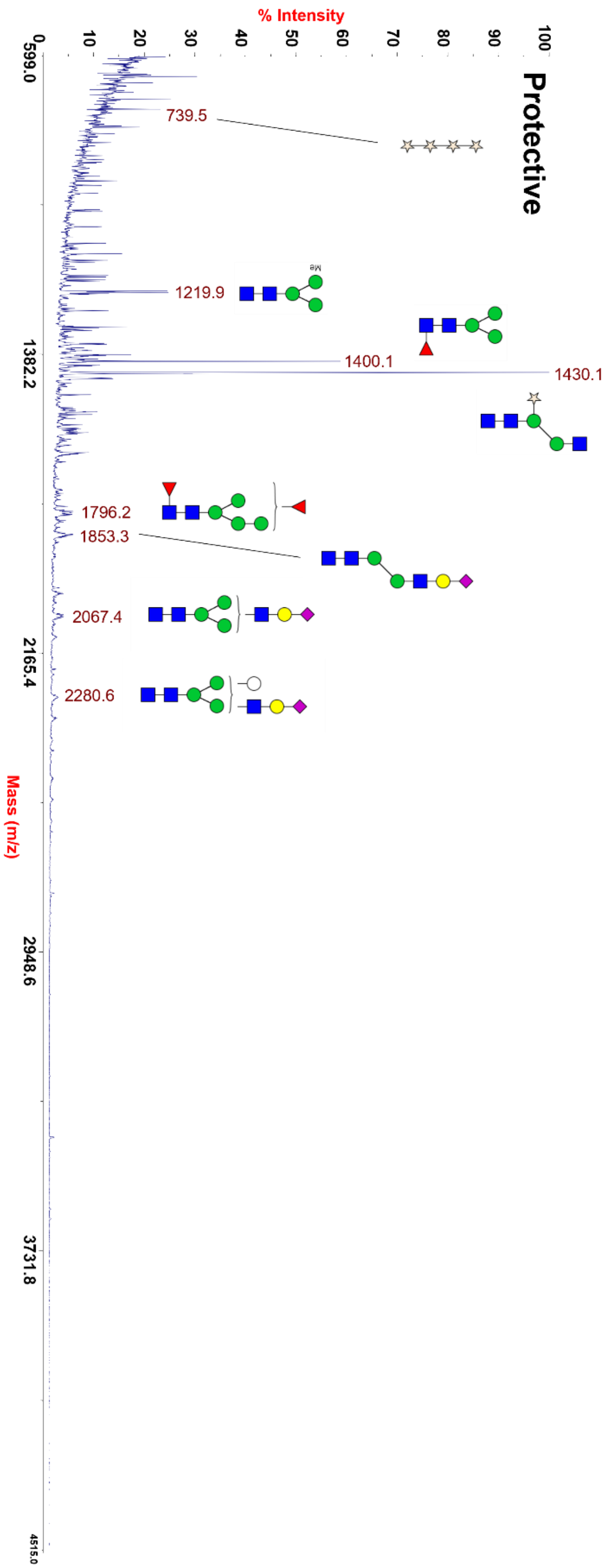

Supplementary Figure 21. Glycomic mass spectrum showing m/z peaks and compositional assignments of extracted *N*-glycans from *C. aspersum* protective mucus samples. Samples were per-deuter-*O*-methylated and adducts shown are Na+.

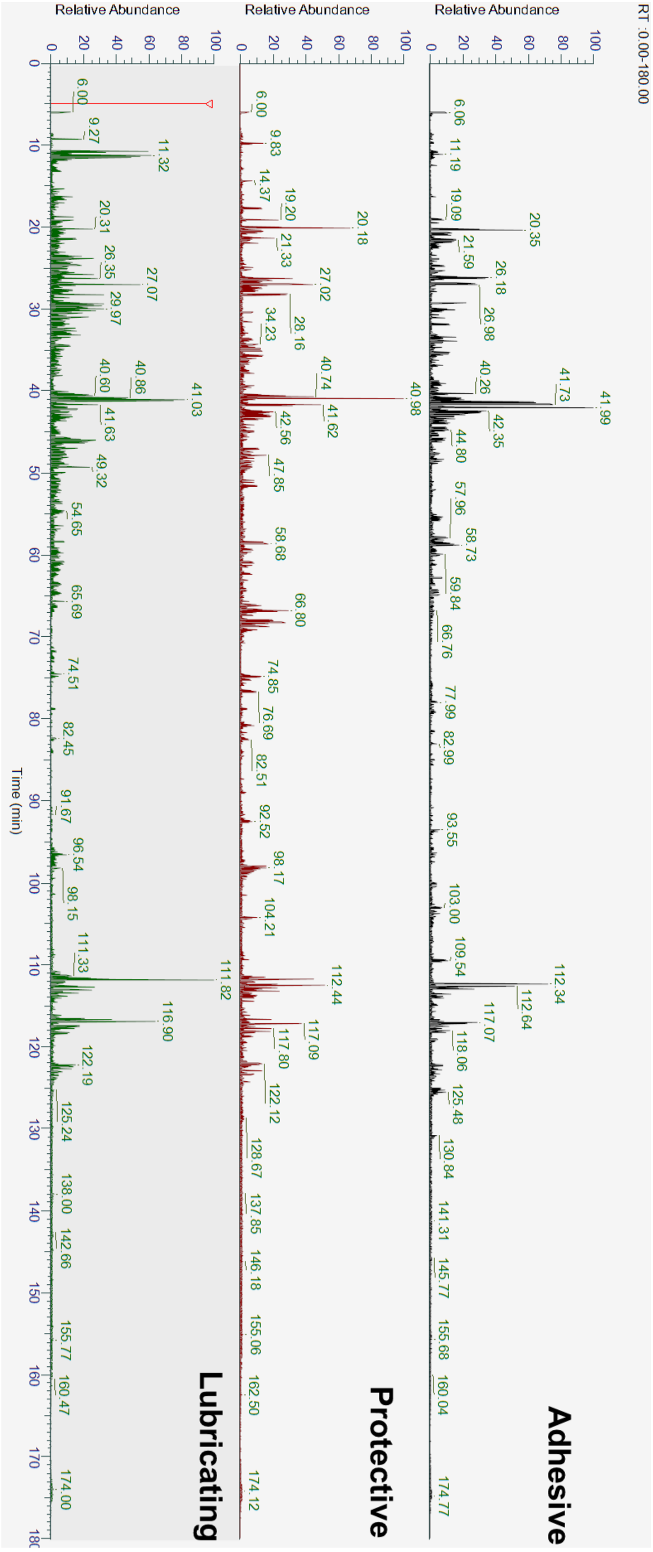

Supplementary Figure 22. Extracted ion chromatograms for m/z 204.0866 (HexNAc signal). Peaks indicate presence of HexNAc at the corresponding time point.

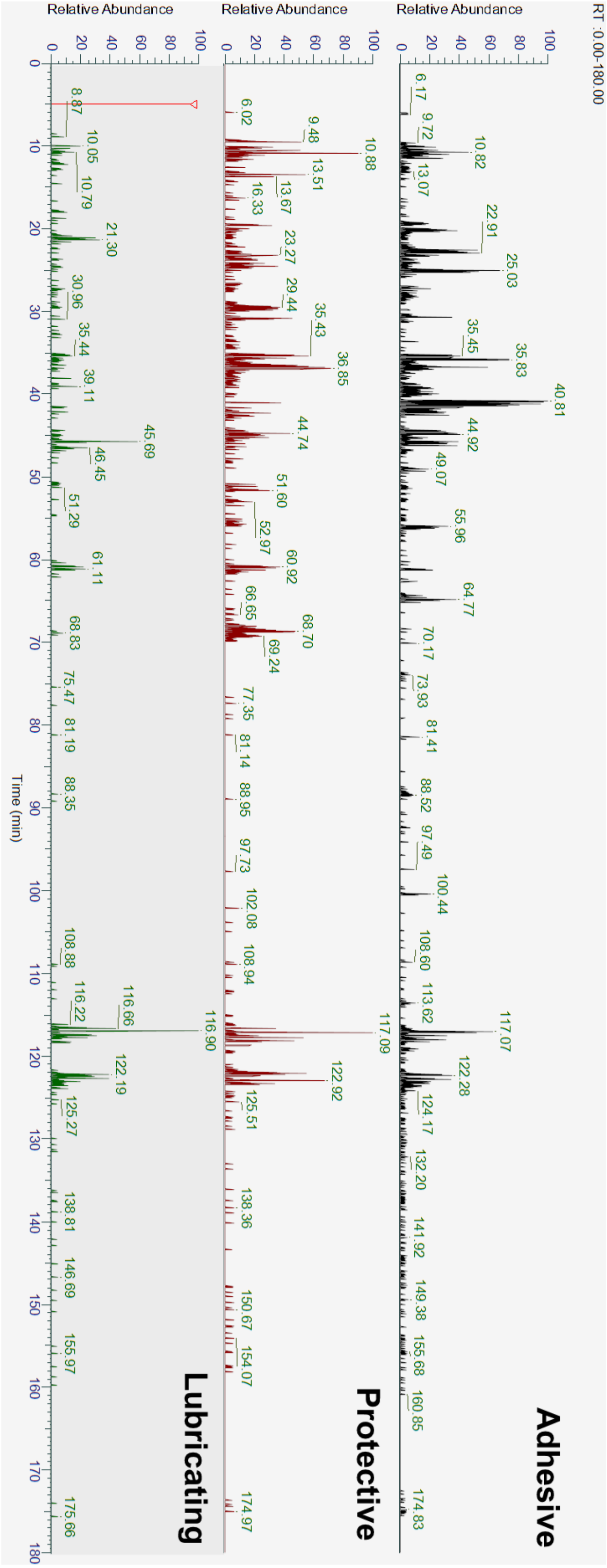

Supplementary Figure 23. Extracted ion chromatograms for m/z 292.1026 (Neu5Ac signal). Peaks indicate presence of Neu5Ac at the corresponding time point.

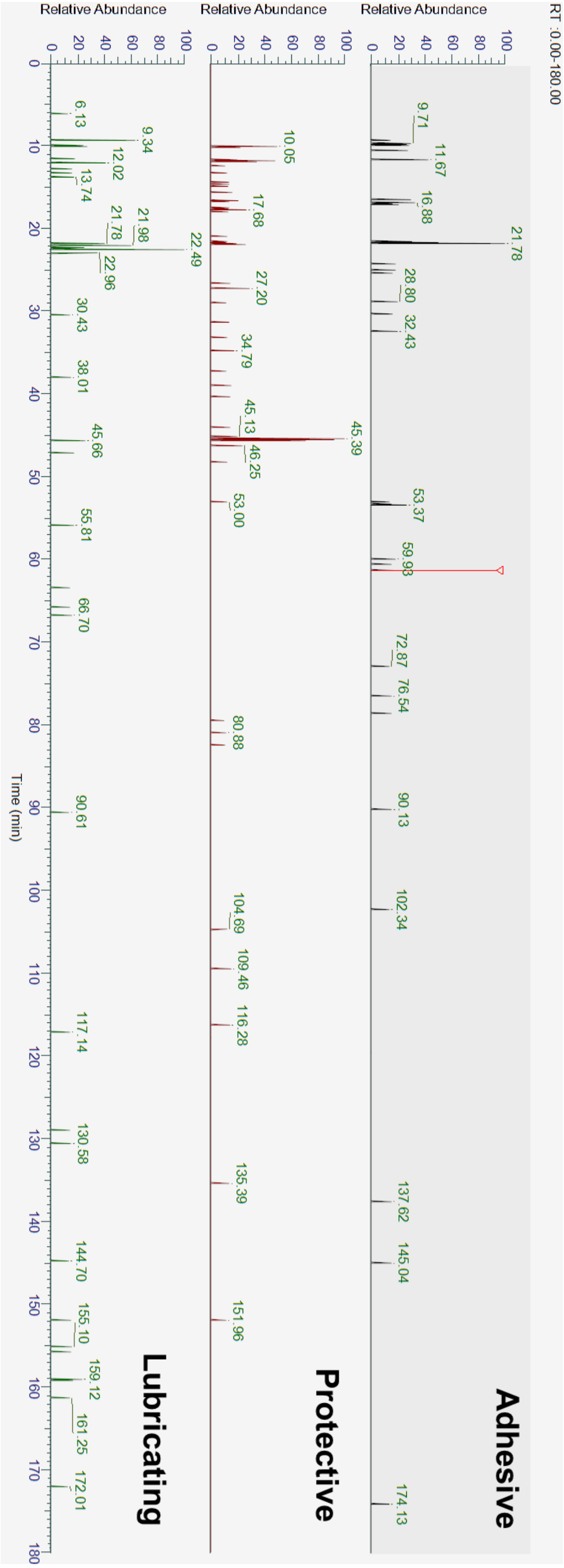

Supplementary Figure 24. Extracted ion chromatograms for m/z 308.0976 (Neu5Gc signal). Peaks indicate presence of Neu5Gc at the corresponding time point.

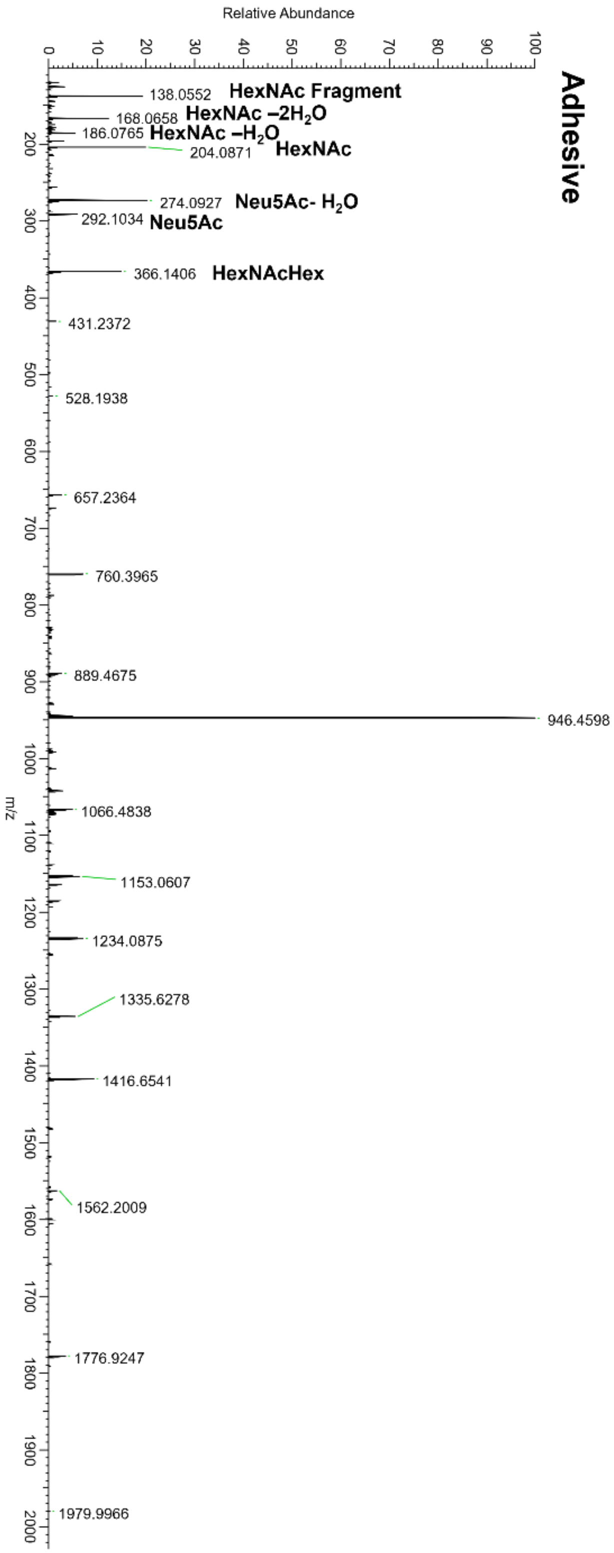

Supplementary Figure 25. Tandem mass spectrum of Neu5Ac-containing glycopeptide from snail adhesive mucus. Spectrum is taken from LC retention time of 40.81 min.

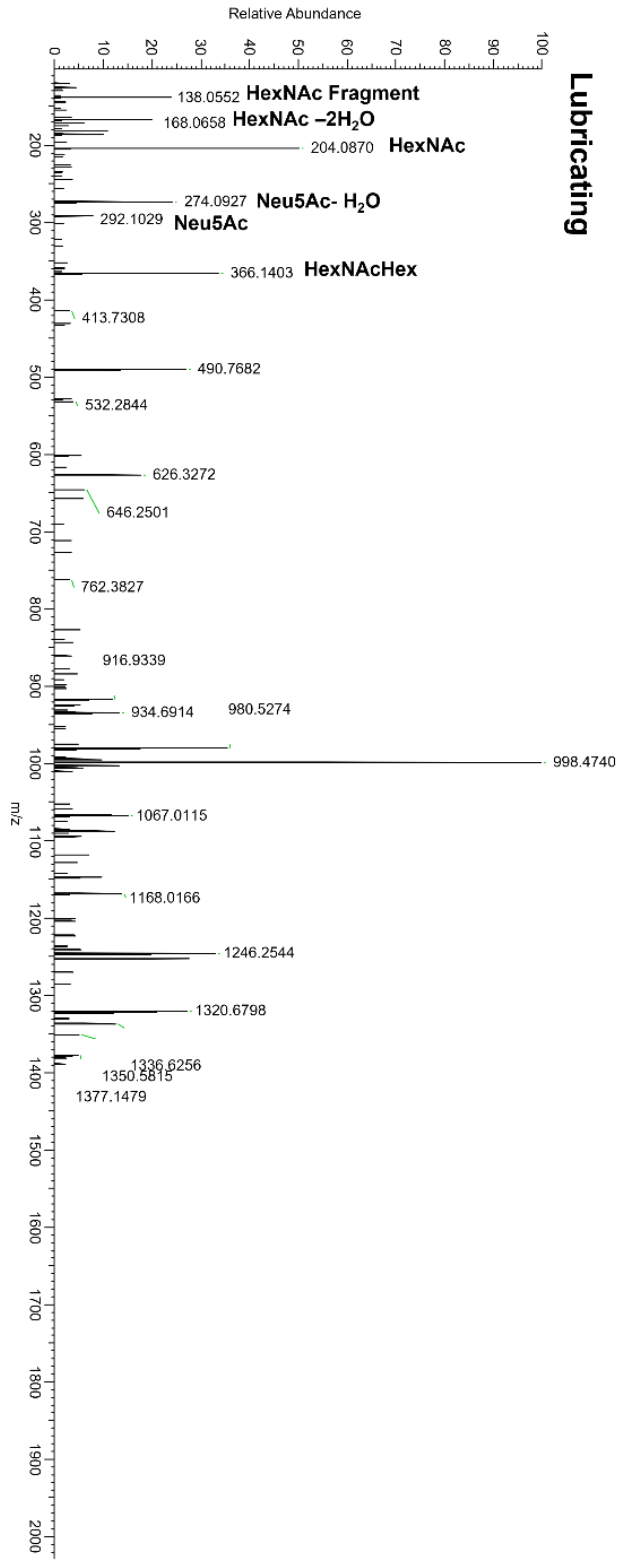

Supplementary Figure 26. Tandem mass spectrum of Neu5Ac-containing glycopeptide from snail lubricating mucus. Spectrum is taken from LC retention time of 44.57 min.

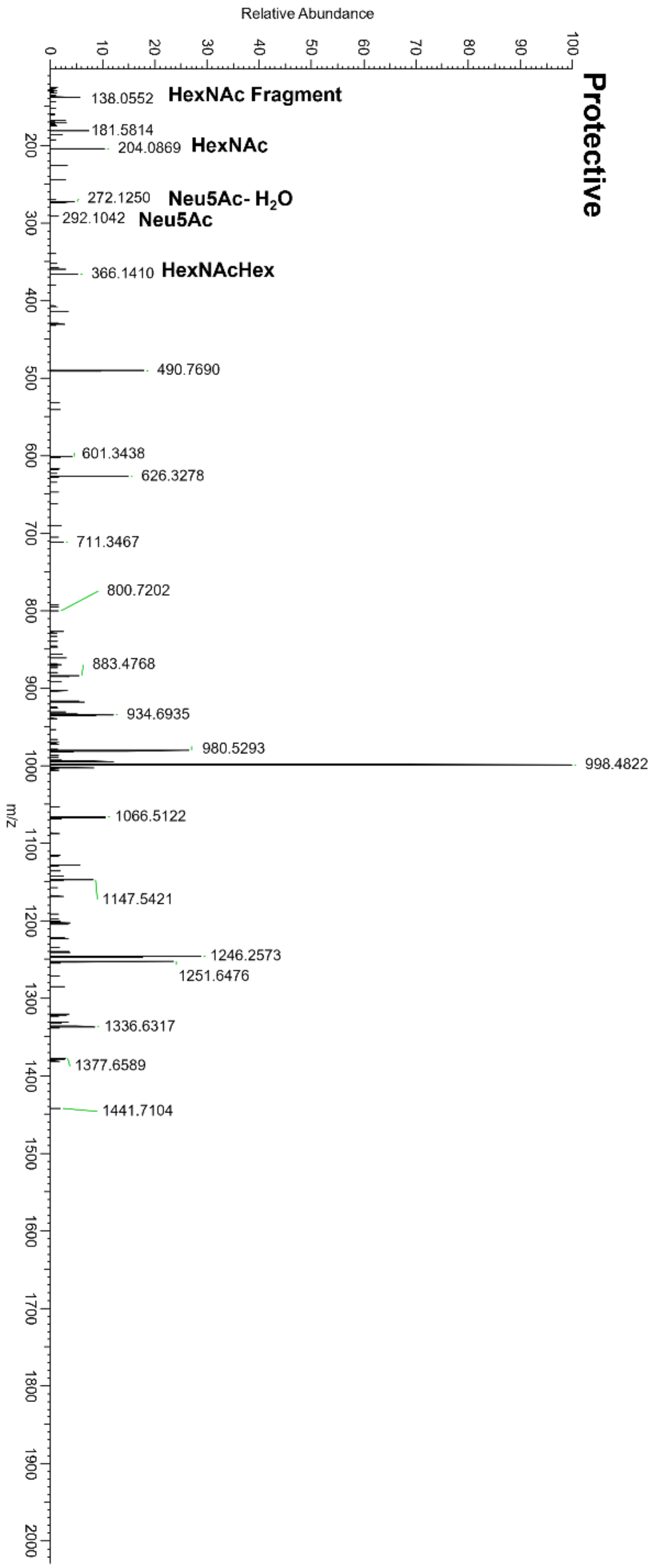

Supplementary Figure 27. Tandem mass spectrum of Neu5Ac-containing glycopeptide from snail protective mucus. Spectrum is taken from LC retention time of 44.89 min.

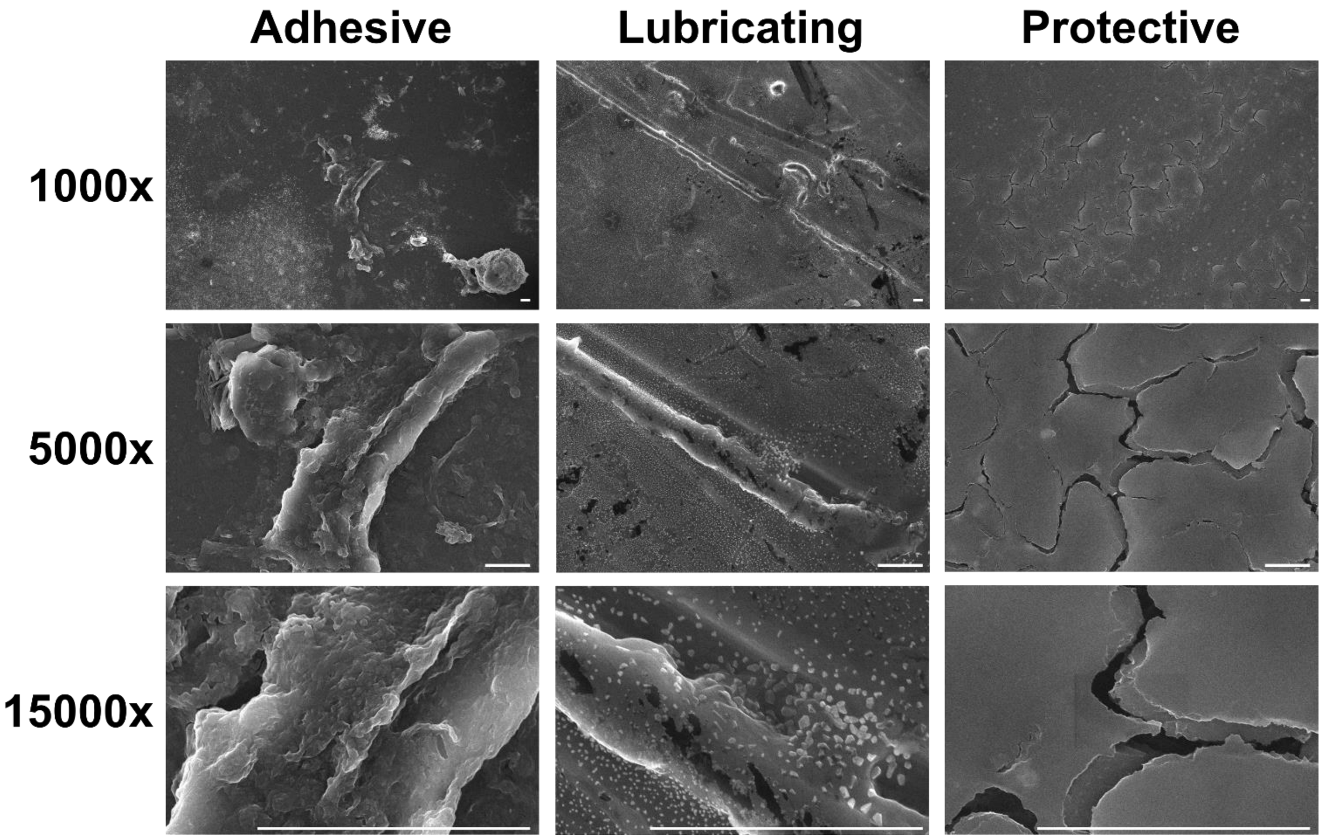

Supplementary Figure 28. SEM imaging of *C. aspersum* adhesive, lubricating, and protective mucus at several magnification levels. Scale bars represent 6 μm. This experiment was repeated independently 3 times with similar results.

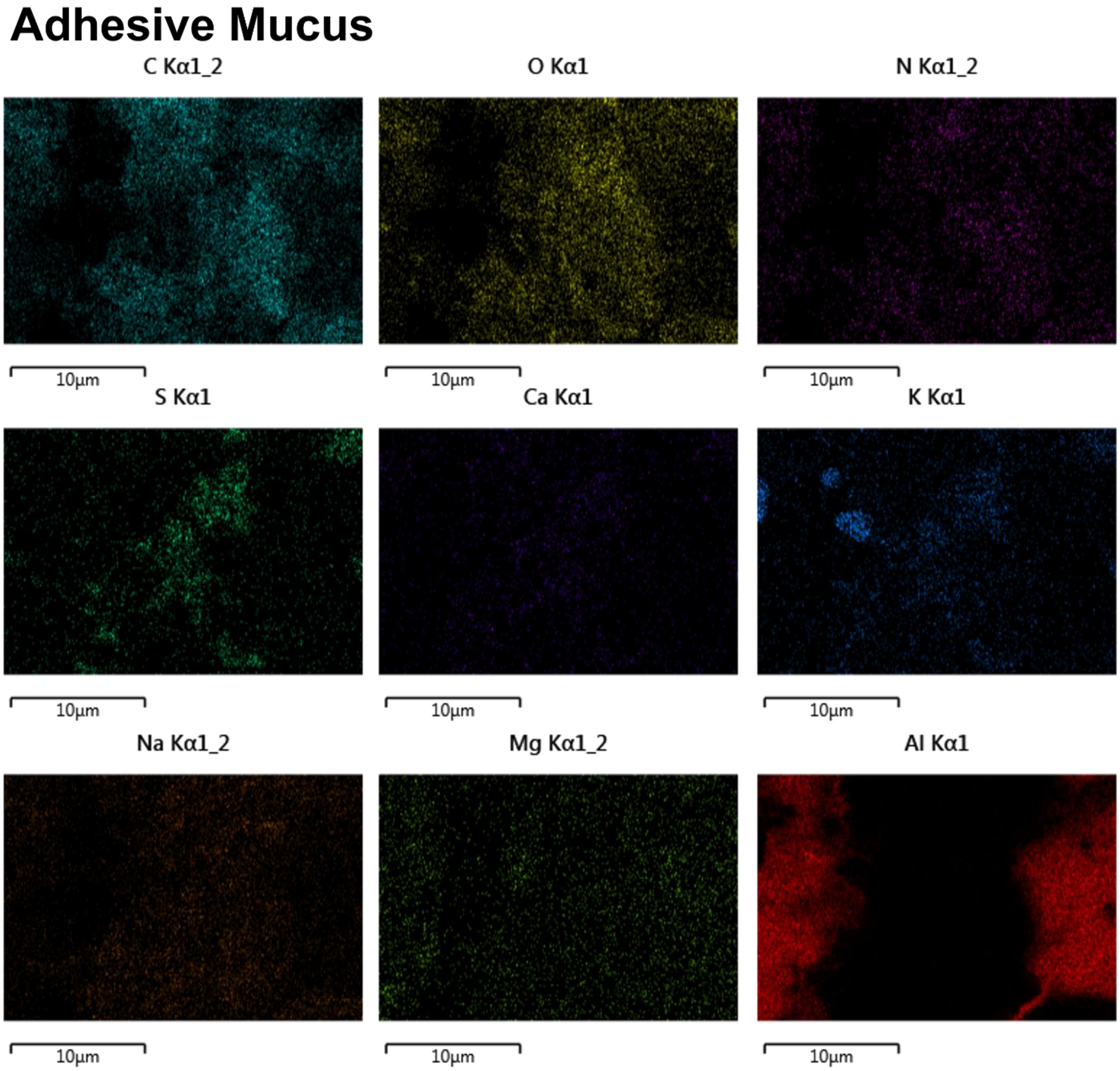

Supplementary Figure 29. SEM EDX overlay images of *C. aspersum* adhesive snail mucus showing localization of detected elements. Scale bars shown. This experiment was repeated independently 3 times with similar results.

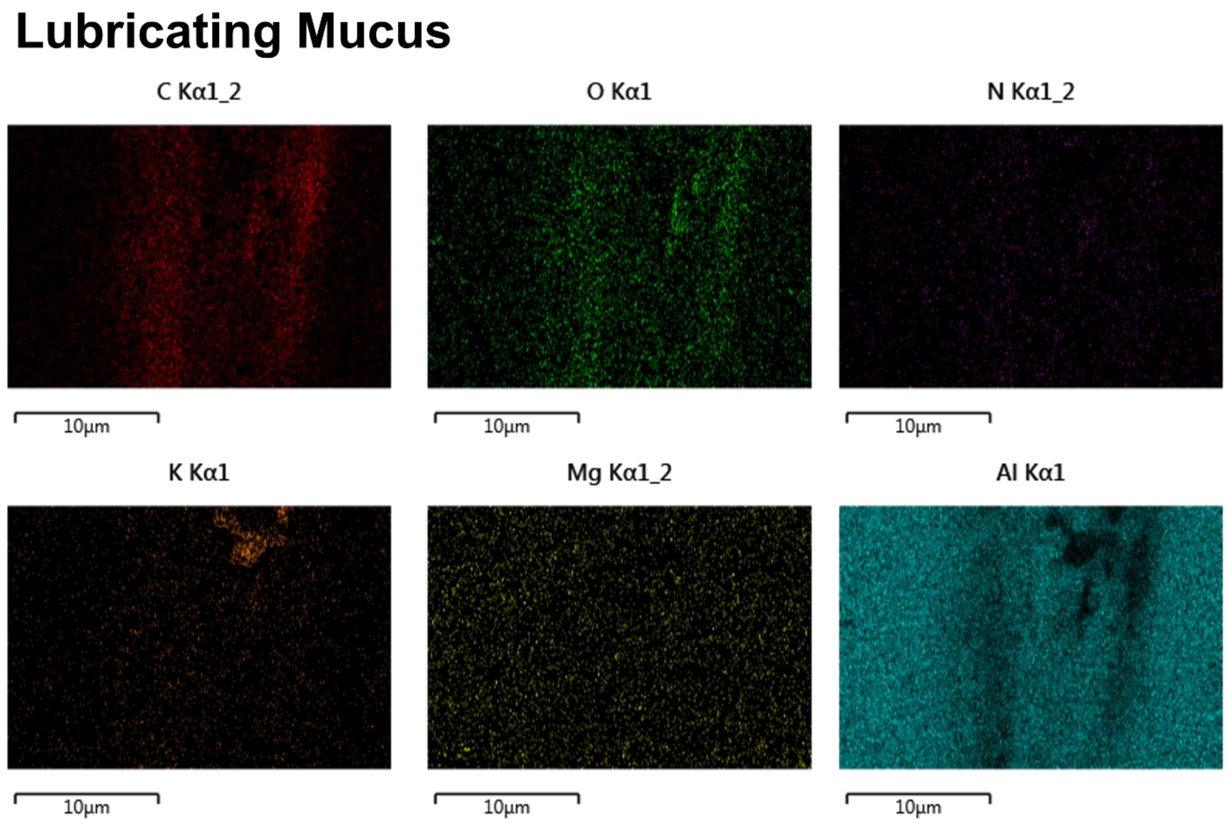

Supplementary Figure 30. SEM EDX overlay images of *C. aspersum* lubricating snail mucus showing localization of detected elements. Scale bars shown. This experiment was repeated independently 3 times with similar results.

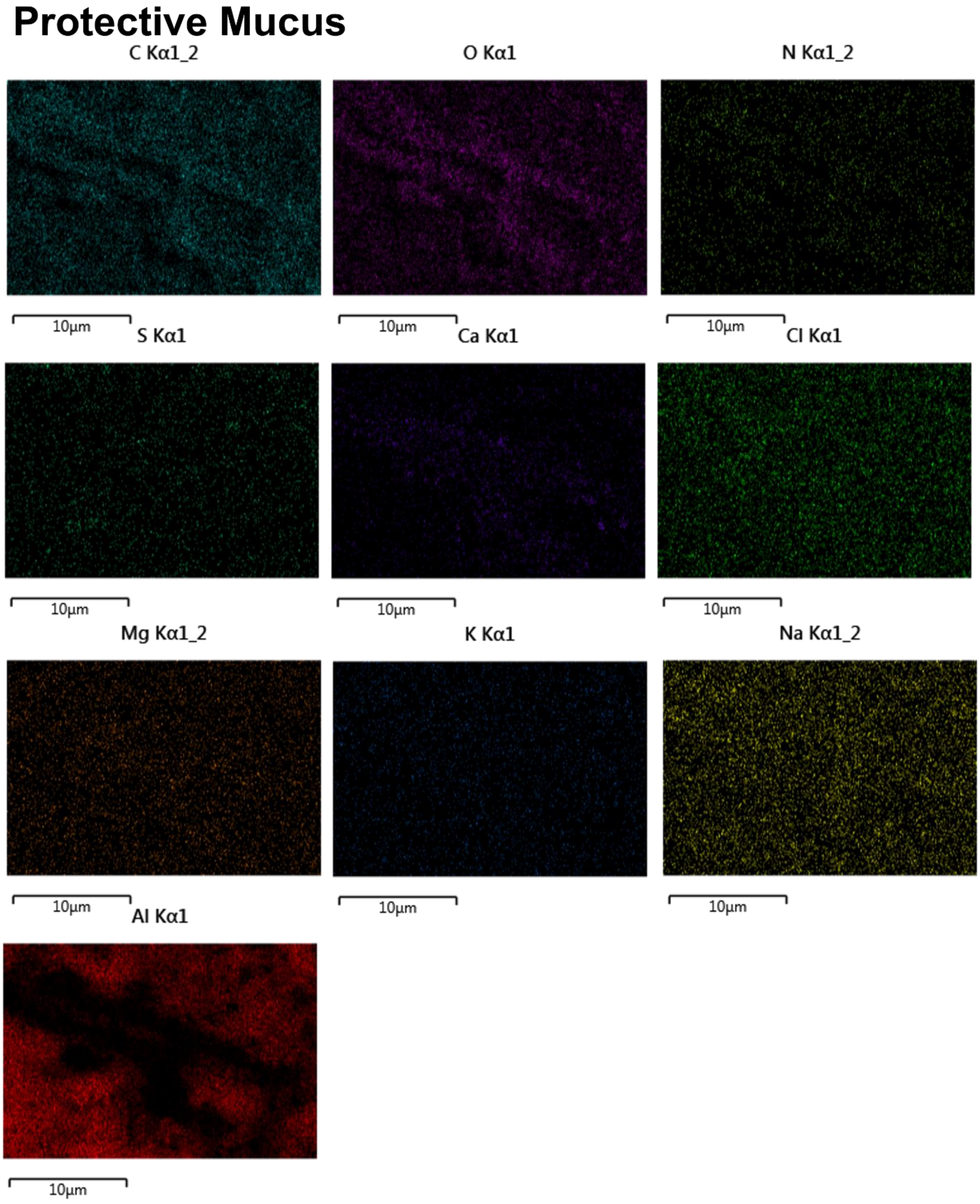

Supplementary Figure 31. SEM EDX overlay images of *C. aspersum* protective snail mucus showing localization of detected elements. Scale bars shown. This experiment was repeated independently 3 times with similar results.

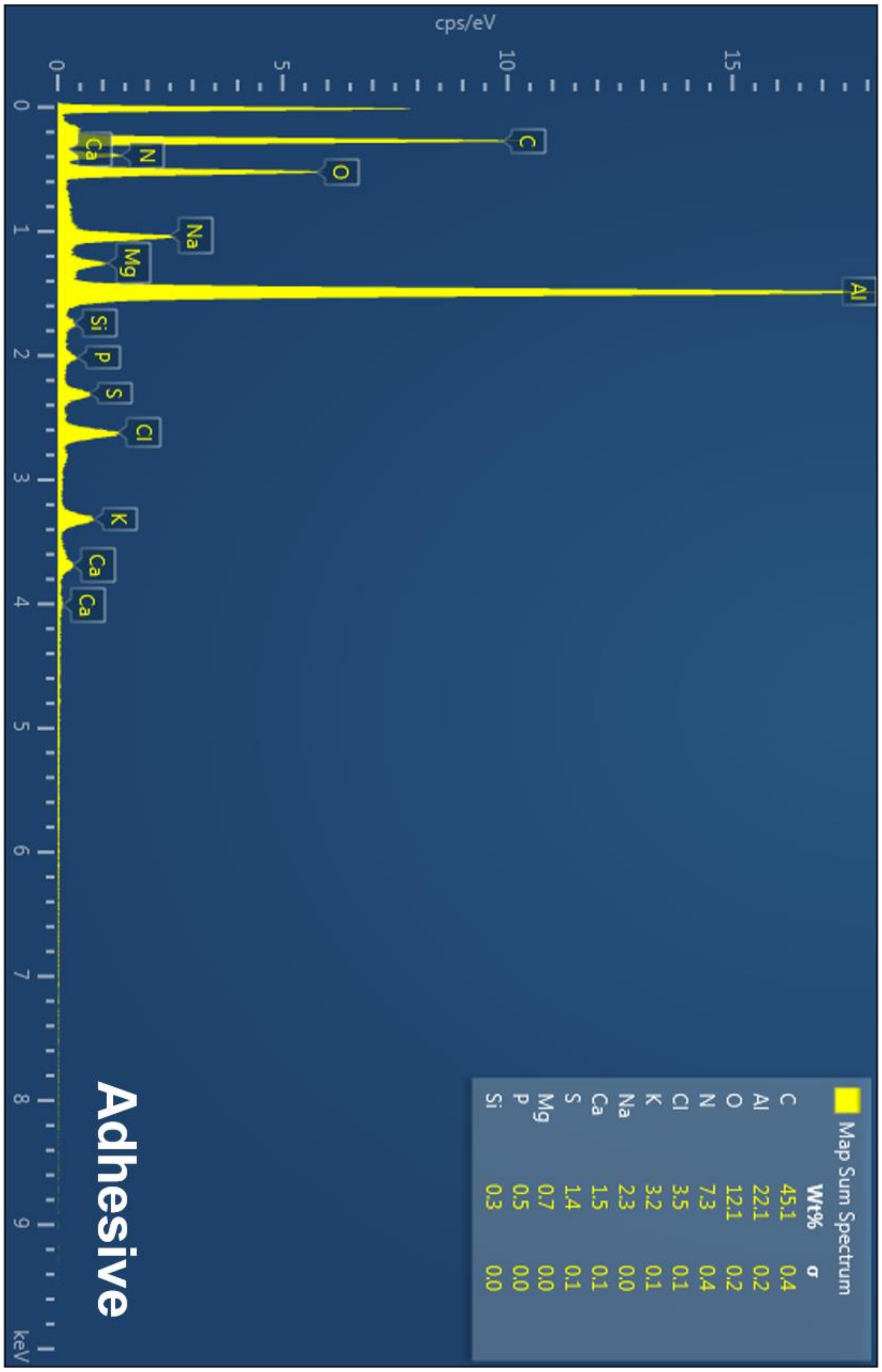

Supplementary Figure 32. Energy Dispersive X-Ray (EDX) spectra showing elemental composition of *C. aspersum* adhesive mucus.

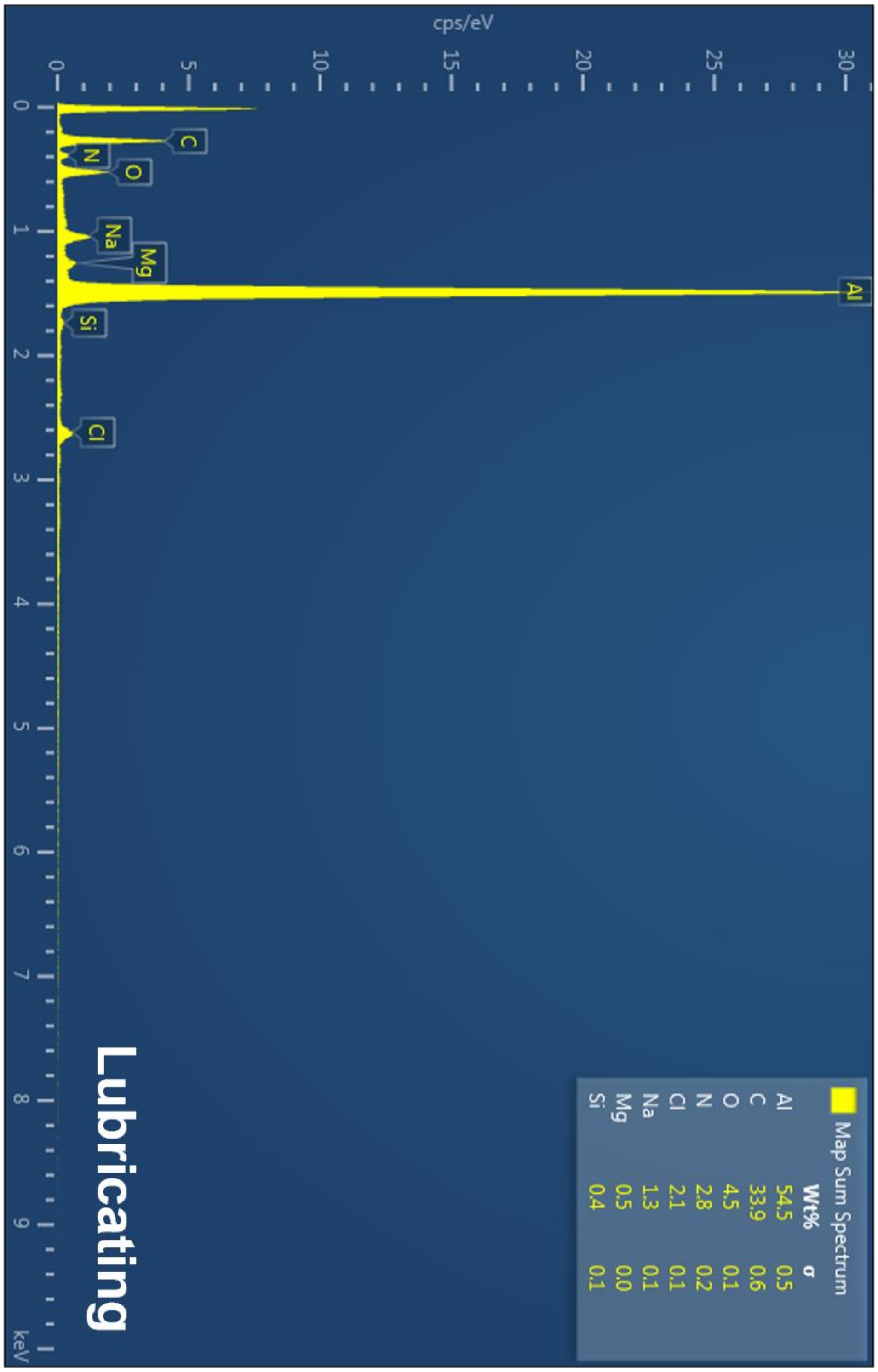

Supplementary Figure 33. Energy Dispersive X-Ray (EDX) spectra showing elemental composition of *C. aspersum* lubricating mucus.

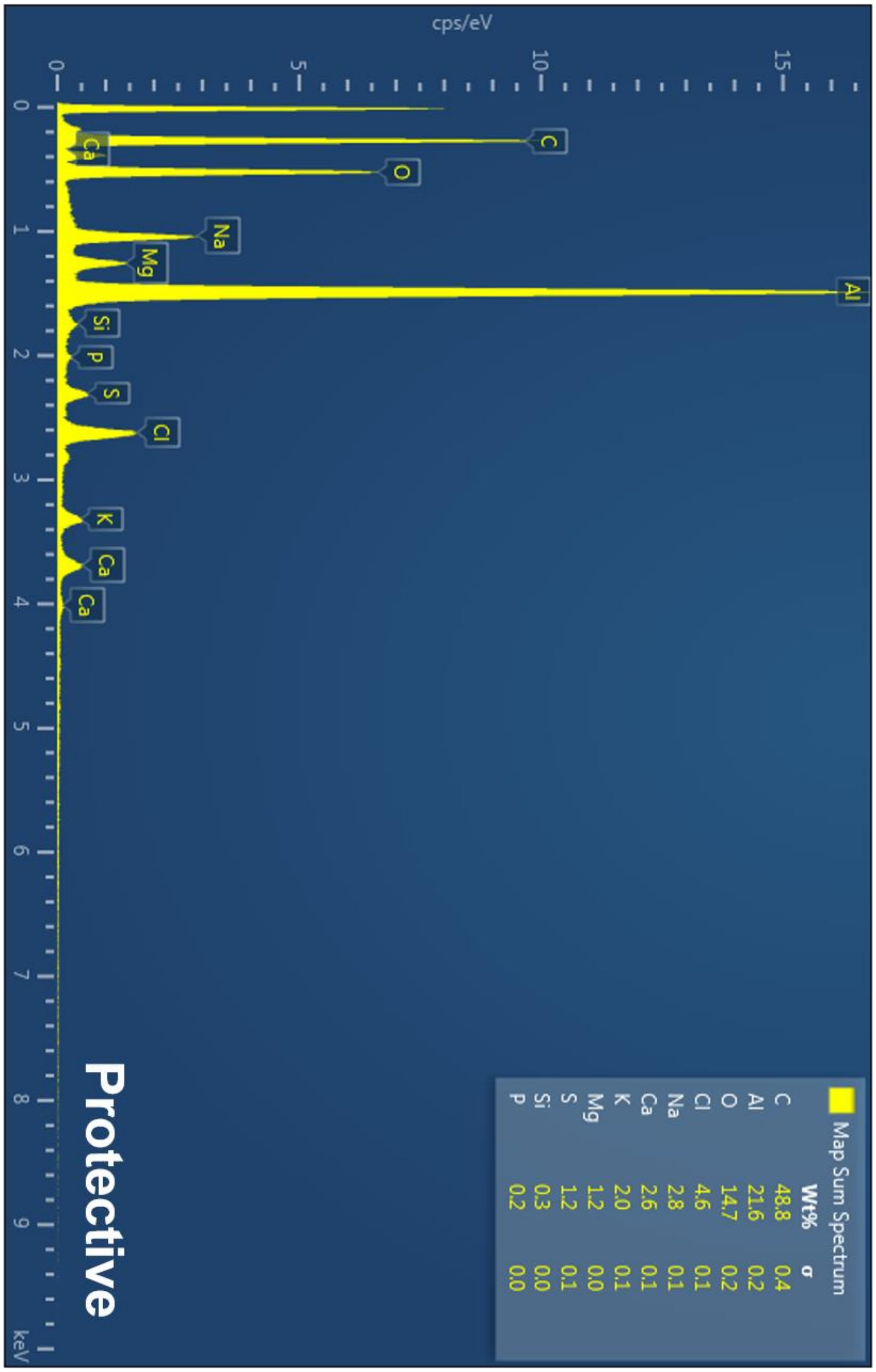

Supplementary Figure 34. Energy Dispersive X-Ray (EDX) spectra showing elemental composition of *C. aspersum* protective mucus.

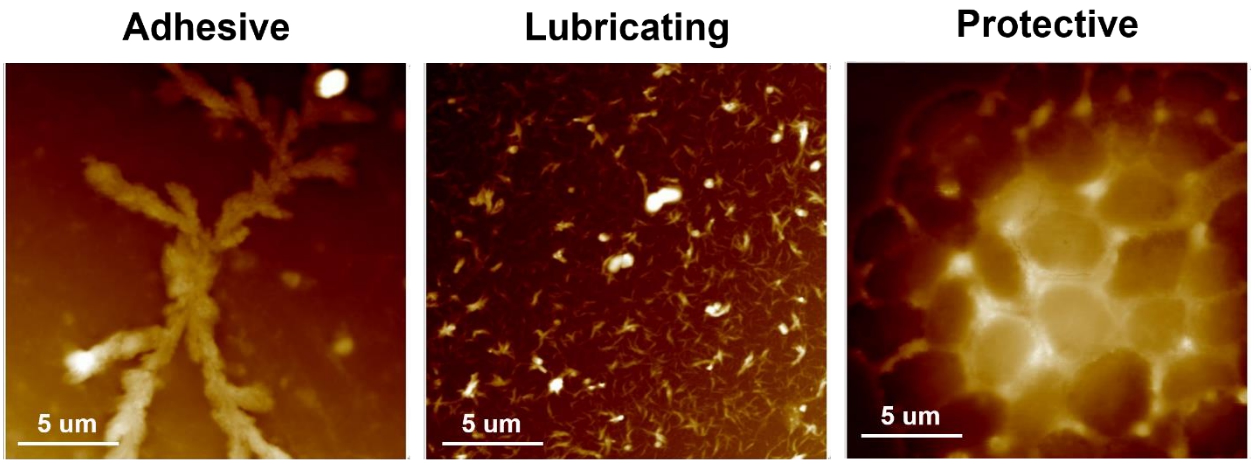

Supplementary Figure 35. AFM topography imaging. Scale bars shown. This experiment was repeated independently 3 times with similar results.

Supplementary Tables

Supplementary Table 1. Concentrations in mg/mL were calculated according to mucus protein standard curve in Supplementary Figure 4. UC stands for ultracentrifugation.

Sample	Protein Content pre-UC (mg)	Protein Content post-UC (mg)	Protein Content post-Dialysis (mg)	% of initial mass
Adhesive	77.2	24.6	2.98	3.86
Lubricating	168.0	29.2	12.4	7.38
Protective	120.0	30.0	5.60	4.67

Supplementary Table 2. Proteins identified in proteomic analysis. Quantification of proteins are based on counts in MS/MS analysis. Corresponding transcriptome accession numbers shown.

Entry	Protein Name	Accession Number	*MW*	Dendrogram Category	Adhesive Counts	Lubricating Counts	Protective Counts
1	Actin1	MM4_TRINITY_DN22220_c0_g1_1	40 kDa	Actins			2
2	Actin2	MM4_TRINITY_DN22220_c0_g3_1	15 kDa	Actins			2
3	Actin3	MM6_TRINITY_DN19769_c0_g1_1	167 kDa	Actins	4	2	5
4	Ankyrin1	MM4_TRINITY_DN21122_c3_g1_1	54 kDa	Glycoproteins			8
5	Ankyrin2	MM3_TRINITY_DN18245_c0_g1_1	34 kDa	Lectins			10
6	Annexin1	MM1_TRINITY_DN12628_c0_g1_1	7 kDa	Gel-forming Mucins			
7	Annexin2	MM3_TRINITY_DN8126_c0_g1_1	14 kDa	Annexins			2
8	Arginase1	MM4_TRINITY_DN16115_c0_g1_1	32 kDa	Arginases		4	
9	ATP-Synthase1	MM6_TRINITY_DN18259_c0_g2_1	58 kDa	ATP Synthases			2
10	CAMP1	MM1_TRINITY_DN11035_c0_g1_1	46 kDa	Gel-forming Mucins			25
11	CAMP2	MM3_TRINITY_DN18384_c0_g2_1	48 kDa	Lectins	2	5	29
12	CAMP3	MM4_TRINITY_DN22211_c1_g4_1	10 kDa	Gel-forming Mucins			11
13	C1q1	MM5_TRINITY_DN15947_c0_g1_1	22 kDa	C1qs			41
14	C1q2	MM4_TRINITY_DN21887_c0_g1_1	84 kDa	Lectins			9
15	C1q3	MM5_TRINITY_DN18999_c2_g1_1	79 kDa	Lectins			10
16	Calmodulin1	MM4_TRINITY_DN21173_c6_g2_1	79 kDa	Ca-binding Proteins		2	5
17	HLectin1	MM3_TRINITY_DN18322_c4_g2_1	28 kDa	Lectins			
18	CD109-1	MM4_TRINITY_DN25046_c0_g1_1	10 kDa	CD-109s		4	4
19	CD109-2	MM6_TRINITY_DN13823_c0_g1_1	53 kDa	CD-109s	3	2	5
20	Collagen1	MM3_TRINITY_DN8024_c0_g1_1	7 kDa	Matrix Proteins	2		
21	Collagen2	MM3_TRINITY_DN17949_c3_g1_1	37 kDa	Gel-forming Mucins		4	
22	Collagen3	MM4_TRINITY_DN21282_c0_g2_1	55 kDa	Gel-forming Mucins		5	
23	Collagen4	MM4_TRINITY_DN21282_c0_g5_1	31 kDa	Matrix Proteins	4	12	3
24	Collagen5	MM5_TRINITY_DN17478_c0_g1_1	65 kDa	Collagens			25
25	Collagen6	MM6_TRINITY_DN18929_c0_g3_1	30 kDa	Collagens	5	9	3
26	Collagen7	MM5_TRINITY_DN19063_c0_g1_1	90 kDa	Collagens	4	3	54
27	Collagen8	MM5_TRINITY_DN19063_c0_g2_1	74 kDa	Collagens		3	50
28	Collagen9	MM6_TRINITY_DN18929_c0_g1_1	57 kDa	Collagens	3	5	5
29	Collagen10	MM4_TRINITY_DN17262_c0_g1_1	18 kDa	Gel-forming Mucins		6	
30	Cystatin1	MM5_TRINITY_DN17535_c0_g1_1	23 kDa	Cystatins		2	24
31	DisulfideIsomerase1	MM1_TRINITY_DN11276_c5_g2_1	14 kDa	Gel-forming Mucins			2
32	EF-Hand1	MM4_TRINITY_DN21829_c0_g1_1	44 kDa	Ca-binding Proteins		5	
33	EF-Hand2	MM6_TRINITY_DN19244_c4_g2_1	37 kDa	Ca-binding Proteins		5	
34	ElongationFactor1	MM4_TRINITY_DN20761_c0_g1_1	62 kDa	Unclustered			5
35	Epiphragmin1	MM6_TRINITY_DN20583_c0_g1_1	7 kDa	Gel-forming Mucins	9		8
36	Epsilon1	MM5_TRINITY_DN5476_c0_g1_1	11 kDa	Unclustered			2
37	Fibrillin1	MM3_TRINITY_DN13586_c0_g1_1	42 kDa	Glycoproteins			7
38	Fibrillin2	MM3_TRINITY_DN17952_c1_g1_1	49 kDa	Matrix Proteins		2	35
39	Fibrinogen1	MM1_TRINITY_DN10983_c2_g2_1	10 kDa	Gel-forming Mucins	8		4
40	Ficolin1	MM1_TRINITY_DN11240_c0_g1_1	41 kDa	Unclustered			7
41	Ficolin2	MM3_TRINITY_DN18425_c0_g1_1	66 kDa	Unclustered		2	9
42	IntermediateFilament1	MM4_TRINITY_DN28955_c0_g1_1	11 kDa	Gel-forming Mucins			3
43	Jagged1	MM2_TRINITY_DN9611_c0_g1_1	31 kDa	Glycoproteins			2
44	Laccase1	MM3_TRINITY_DN15913_c0_g1_1	54 kDa	Laccases			9
45	Novel2	MM3_TRINITY_DN17597_c0_g1_1	24 kDa	Lectins			3
46	Novel3	MM3_TRINITY_DN18249_c5_g4_1	64 kDa	Glycoproteins			13
47	Novel4	MM4_TRINITY_DN21224_c1_g1_1	9 kDa	Matrix Proteins	2	5	
48	Novel5	MM4_TRINITY_DN21844_c0_g1_1	14 kDa	Gel-forming Mucins	12		
49	Novel6	MM6_TRINITY_DN21806_c0_g1_1	9 kDa	Matrix Proteins	37	2	19
50	ProlylIsomerase1	MM6_TRINITY_DN18216_c0_g1_1	63 kDa	Prolyl Isomers			2
51	Proteoglycan1	MM1_TRINITY_DN9148_c0_g1_1	32 kDa	Glycoproteins		3	3
52	Serpin1	MM5_TRINITY_DN18792_c2_g1_1	77 kDa	Serpins	6		9
53	Snail1	MM1_TRINITY_DN11259_c0_g2_1	67 kDa	Glycoproteins			4
54	Snail2	MM1_TRINITY_DN10906_c0_g2_1	30 kDa	Lectins			2
55	Snail3	MM2_TRINITY_DN9959_c0_g1_1	40 kDa	Lectins			3
56	Snail4	MM5_TRINITY_DN16950_c0_g1_1	18 kDa	Mucins	4	10	3
57	Snail5	MM5_TRINITY_DN18004_c6_g1_1	77 kDa	Glycoproteins	22	2	12
58	Snail6	MM4_TRINITY_DN19690_c0_g1_1	43 kDa	Gel-forming Mucins			2
59	Snail7	MM4_TRINITY_DN21671_c4_g6_1	25 kDa	Glycoproteins			3
60	Snail8	MM6_TRINITY_DN19636_c3_g1_1	70 kDa	Lectins		4	22
61	Snail9	MM6_TRINITY_DN19640_c2_g1_1	112 kDa	Glycoproteins			15
62	Snail10	MM4_TRINITY_DN21618_c2_g4_1	29 kDa	Lectins			10
63	Snail11	MM5_TRINITY_DN19011_c0_g2_1	62 kDa	Lectins			3
64	Snail12	MM6_TRINITY_DN19602_c1_g1_1	51 kDa	Lectins			3
65	Spondin1	MM4_TRINITY_DN481_c0_g1_1	64 kDa	Glycoproteins		5	
66	Tenascin1	MM3_TRINITY_DN18052_c0_g1_1	76 kDa	Glycoproteins	26		10
67	Tenascin2	MM2_TRINITY_DN10241_c6_g1_1	76 kDa	Glycoproteins	26		10
68	Ficolin3	MM4_TRINITY_DN22280_c4_g1_1	87 kDa	Glycoproteins			12
69	Novel1	MM3_TRINITY_DN18425_c0_g2_1	14 kDa	CD-109s		2	6
70	TranslationInitiationFactor1	MM1_TRINITY_DN11217_c5_g1_1	9 kDa	Unclustered			8
71	Tyrosinase1	MM6_TRINITY_DN19765_c0_g2_1	72 kDa	Tyrosinases	3		

Supplementary Table 3. Specific proteins found in *C. aspersum* snail mucus and their known functions. Snail, Novel, and Other categories not included.

Protein	Group	Function
Laccase	Enzyme	Catalyzes oxidation of phenolic compounds
Arginase		Catalyzes formation of urea from arginine
Prolyl Isomerase		Interconverts cis-trans isomers of proline peptide bonds
ATP Synthase		Produces ATP from ADP
Tyrosinase		Catalzyes formation of DOPA from tyrosine and melanins
Disulfide Isomerase		Catalyzes formation and breakage of disulfide bonds
CD109	Inhibitor	Serine protease inhibitor
Cystatin		Cysteine protease inhibitor
Serpin		Serine protease inhibitor
C1q	Lectin	Lectin, binds with serine proteases
H-Type Lectin		GalNAc-binding lectin
Annexin	Ion-Binding	Calcium ion regulation
Calmodulin		Calcium-binding messenger protein
EF-Hand		Calcium-binding signaling protein
Proteoglycan	Matrix	Heavily glycosylated protein found in the extracellular matrix
Jagged-1		Cell-surface signalling protein
Fibrillin		Secreted glycoprotein that forms elastic fibers
Spondin		Secreted glycoprotein involved in extracellular matrix
Collagen		Main structural protein of the extracellular matrix
Actin		Cytoskeleton structural protein
Tenascin		Extracellular matrix glycoprotein
Epiphragmin	Network	Main protein component of snail epiphragm (adhesive seal)
Ficolin		Oligomeric lectins with collagen- and fibrinogen-like domains
Mucin		Main glycoprotein component of mucus
Fibrinogen		Glycoproteins that form oligomeric networks

Supplementary Table 4. Proposed functions for uncharacterized proteins based on phylogenetic analysis. "Snail" refers to proteins without any determinable function but had structural similarity to uncharacterized proteins previously found in snails. "Novel" indicates the protein had no similarity to any known proteins in the NCBI nor PFAM databases.

Protein	Clade	Putative Function
Snail1	Mollusk Glycoproteins	Proteoglycan
Snail2	Lectins	Agglutinin
Snail3	Lectins	Agglutinin
Snail4	Mollusk Mucins	Mucin
Snail5	Mollusk Glycoproteins	Proteoglycan
Snail6	Gel-Forming Mucins	Mucin
Snail7	Mollusk Glycoproteins	Jagged-1
Snail8	Lectins	Lectin
Snail9	Mollusk Glycoproteins	Proteoglycan
Snail10	Lectins	H-Lectin
Snail11	Lectins	Lectin
Snail12	Lectins	Lectin
Novel1	CD109s	CD109
Novel2	Lectins	Agglutinin
Novel3	Mollusk Glycoproteins	Proteoglycan
Novel4	Matrix Proteins	Fibrillin
Novel5	Gel-Forming Mucins	Mucin
Novel6	Matrix Proteins	Collagen

Supplementary Table 5. Proteomic quantification of proteins identified in the 260 kDa band of the SDS-PAGE gels shown in Figures S9 – 11. Percent abundance refers to percentage of each protein relative to total protein per sample within the band. Proteins of abundance greater than 5 % of total protein content are shown.

Protein	Accession	Mw	Percent Abundance		
			Adhesive	Lubricating	Protective
Collagen5	MM5_TRINITY_DN17478_c0_g1	65.0	0.0	0.0	5.9
Collagen7	MM5_TRINITY_DN19063_c0_g1	89.8	0.0	0.0	11.8
Novel4	MM4_TRINITY_DN21224_c1_g1	9.4	20.0	33.3	7.8
Novel4A	MM4_TRINITY_DN21224_c6_g1	46.3	0.0	11.1	7.8
Novel7	MM3_TRINITY_DN18065_c5_g2	31.1	40.0	33.3	9.8
Novel8	MM5_TRINITY_DN17583_c0_g1	46.6	20.0	11.1	2.0

Supplementary Table 6. Glycoproteomic identification of proteins found in proteomic analysis of the 260 kDa band of the SDS-PAGE gels shown in Figures S9 – 11. Proteins from Table S5 not included here did not present detectable glycopeptides.

Protein	Peptide	Glycan	Sample
Novel4	P.KDQIS[+365.13220]DILK.K	HexNAc(1)Hex(1)	Protective
Collagen5	Q.IS[+689.23784][+14.01570]NKDVR.F	HexNAc(1)Hex(3) + Me	Protective
	G.LGFELQAIAS[+203.07937]NYK.N	HexNAc(1)	Adhesive
	A.IAS[+689.23784][+14.01570]NYK.N	HexNAc(1)Hex(3) + Me	Protective
	R.AGS[+527.18502]VINPK.E	HexNAc(1)Hex(2)	Protective
	R.AGSVINPKET[+527.18502][+28.03140]NK.C	HexNAc(1)Hex(2) + 2Me	Adhesive
	P.KET[+527.18502][+28.03140]NK.C	HexNAc(1)Hex(2) + 2Me	Lubricating
	R.GALFGGLLFAT[+568.21157]EVQK.L	HexNAc(2)Hex(1)	Protective
	G.GLLFAT[+527.18502][+14.01570]EVQK.L	HexNAc(1)Hex(2) + Me	Adhesive
	P.PS[+365.13220]AKAAADDLK.S	HexNAc(1)Hex(1)	Protective
	K.AAADDLKS[+527.18502][+28.03140]Q.G	HexNAc(1)Hex(2) + 2Me	Protective
Collagen7	K.NT[+527.18502][+14.01570]FIK.A	HexNAc(1)Hex(2) + Me	Lubricating

Supplementary Table 7. Amino acid sequences of CAMPs.

Protein	Sequence
CAMP1	NYLRFRIGISGFICGVLLVVVSVTTIGQGERIVFNAKPTTISPGVT<br>PELTVRCGLEDDGNSGVSRVNSIIIRTVDGSVQKEVARIAYRQAAT<br>GGFSTEGASVTGDLSNKAGYLQITWPSPRHGLAGQYNCDIAALATV<br>GDIVKFKSSIRVVSTGKIADLSLSPAFWSAQVKMMAVQTRSNANAQ<br>KTLRHRKRLGTVKGNLILRKRMIRRISAAVQFTTKRALFRSEILIL<br>GENHKLTKRALFRAEILILGENKKKGRRSKSASQLPMPGTRCWSFL<br>PWFWMRIIQESSDTELESILRQEPAGGHFSRGSGCGYKKPQIQNWN<br>QGCHTHGFQGCLWHLVGHNPVSYCPVGQCGITLSQPQTYTCAIIDS<br>NNRHQSGFWLVSLPYVFVHQYQMYVPTCIVIFSTIQLWFLSSKX
CAMP2	HLSHTDTQYMCVLGSMKEDSISIPIHGCGVFYLQTDRTIVGLSVVC<br>CLLLSLQQVKVKESYSTQSQRLSLQQVKVKESSSRQHQQPSPREHQ<br>SFVFSATPITISPGVTPELTVRCGLEDDGNSGVSRVNSIIIRTVDG<br>SVQKEVARIAYRQAATGGFSTEGASVTGDLSNKAGYLQITWPSPRH<br>GLAGQYNCDIAALATVGDIVKFKSSIRVVSTGKIADLSLSPAFWSA<br>QVKMMAVQTRSNANAQKTLRHRKRLGTVKGNLILRKRMIRRISAAV<br>QFTTKRALFRAAILILGENKKIRDSDSWGEPLKARETVVEVRFSTS<br>YARNPLLVVSPVVLDADNTTPGTRWWSFLPWFWMWLIQKTSDTELE<br>SSDTELESKIIRYRIRIRDVTPTGFKVVCGTWWDTILYRIDVRWVS<br>NVARCLSHRHTHVQS
CAMP3	GDLSDKAGYLQVTWPSPEHGLAGQYTCDIVAVAESGDNIKFKSSIQ<br>VVSTGKNADSSDSKQCQCTTDIEALKKAVRDSQGKFDSLEKTVNDL<br>KTSX

Supplementary Table 8. Glycoproteomic analysis of CAMPs. Example tandem MS/MS spectrum of CAMP2, glycopeptide 3 is shown with b ions, y ions, and glycorelated fragment labelled.

Protein	Peptide	Glycan
CAMP1	R.ALFRS[+673.24293]E.I	HexNAc(1)Hex(2)Fuc(1)
CAMP2	C.GVFYLQT[+527.18502][+14.01570]DR.T*	HexNAc(1)Hex(2) + Me
	R.ET[+673.24293]VVEVR.F	HexNAc(1)Hex(2)Fuc(1)
	A.T[+406.15875]VGDIVK.F	HexNAc(2)
	L.QT[+203.07937]DR.T	HexNAc(1)
	V.LGS[+203.07937]M[+15.99492]K.E**	HexNAc(1)
CAMP3	K.AVRDS[+527.18502]QGK.F	HexNAc(1)Hex(2)
	R.DS[+527.18502][+28.03140]QGK.F*	HexNAc(1)Hex(2) + 2Me

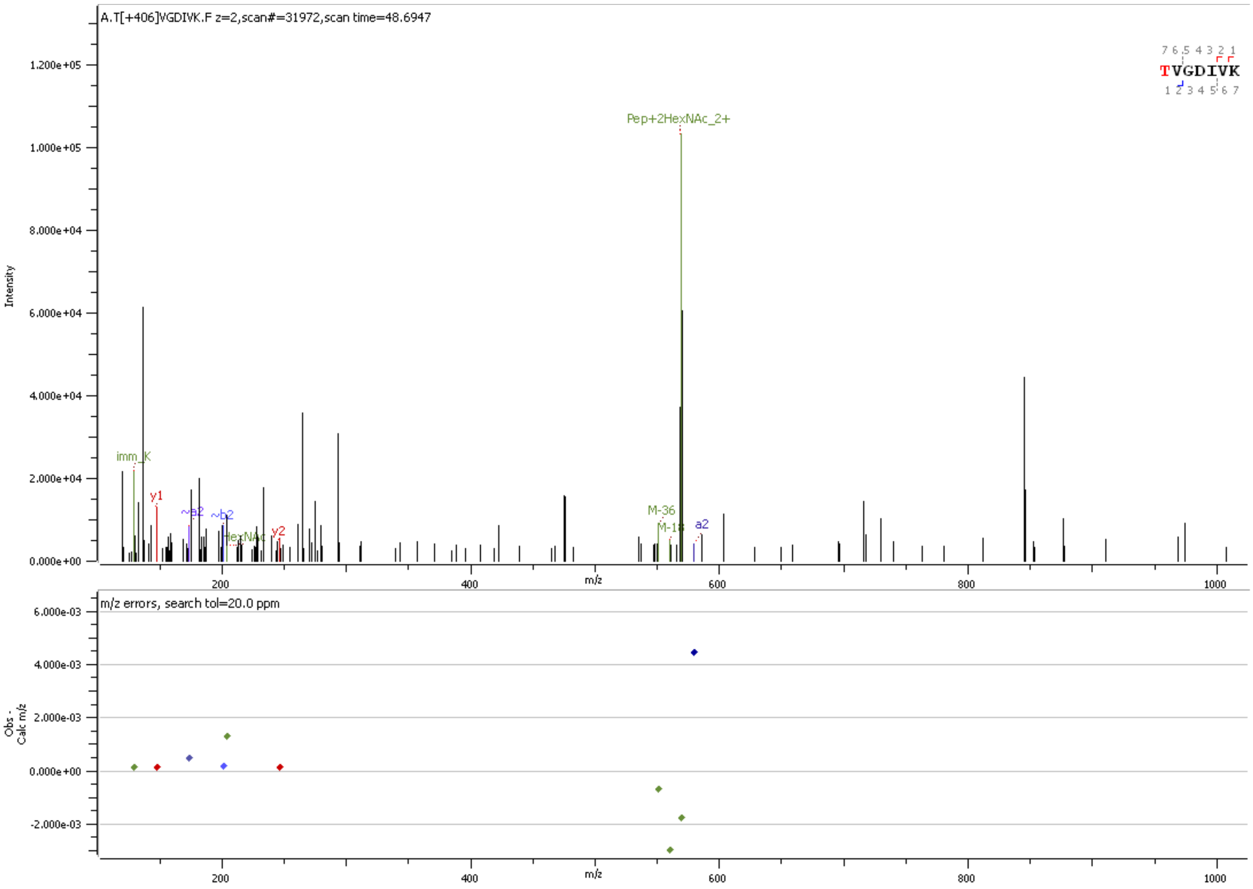

*****Shift of S/T[+14.01570] and [28.03140] indicates methylated and demethylated glycans, respectively, O-linked to serine or threonine.*

*****Shift of M[+15.99492] indicates methionine oxidation to methionine sulfoxide*

Supplementary Table 9. *O*-Glycans extracted from adhesive, lubricating, and protective, *C. aspersum* snail mucus proteins that were detected via glycomic mass spectrometry analysis. Relative percentage refers to the ratio of the area of each individual glycan MS peak to the total area of all glycan peaks for a given experiment. Samples were per-deuter-*O*-methylated and adducts shown are Na+.

Sample	Glycan m/z	Monosaccharide Composition	Proposed Structure	Relative Percentage
Adhesive	561.5	Gal1GalNAc1	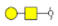	48.22%
	768.7	Gal2GalNAc1 + 2Me	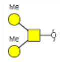	14.56%
	771.7	Gal2GalNAc1 + 1Me	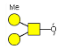	14.66%
	774.7	Gal2GalNAc1	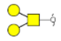	9.56%
	981.9	Gal3GalNAc1 + 1Me	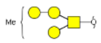	5.30%
	1192.1	NeuAc1Gal1HexNAc1GalNAc1	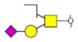	5.45%
	1552.4	NeuAc1Fuc2Gal1HexNAc1GalNAc1	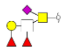	2.25%
Lubricating	768.7	Gal2GalNAc1 + 2Me	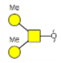	14.56%
	954.97	Fuc1Gal2GalNAc1	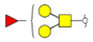	5.30%
	982.8	Gal3GalNAc1 + 1Me	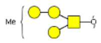	5.30%
Protective	534.5	Gal1GalNAc1		45.10%
	738.8	Gal2GalNAc1 + 2Me	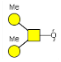	48.90%
	942.8	Fuc1Gal2GalNAc1	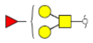	2.80%
	983.9	Gal3GalNAc1 + 1Me	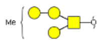	2.20%

Supplementary Table 10. *N*-Glycans extracted from adhesive, lubricating, and protective, *C. aspersum* snail mucus proteins that were detected via glycomic mass spectrometry analysis. Relative percentage refers to the ratio of the area of each individual glycan MS peak to the total area of all glycan peaks for a given experiment. Samples were per-deuter-*O*-methylated and adducts shown are Na+.

Sample	Glycan m/z	Monosaccharide Composition	Proposed Structure	Relative Percentage
Adhesive	1382.96	Man3GlcNAc2Xyl1 + 2 Me	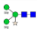	30%
	1862.33	Man6GlcNAc2	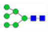	8%
	2075.46	Man7GlcNAc2	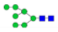	9%
	2288.6	Man8GlcNAc2	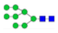	10%
	2419.69	HexNAc2GlcNAc2Man3GlcNAc2Fuc1	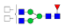	4%
	2501.73	Man9GlcNAc2	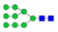	8%
	2632.81	Gal1GlcNAc4Man3GlcNAc2Fuc1	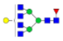	4%
	2680.8	NeuAc1Gal1GlcNAc2Man3GlcNAc2Fuc2	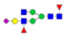	3%
	3059.06	Gal3GlcNAc4Man3GlcNAc2Fuc1	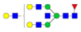	3%
	3138.14	Fuc1Hex1GlcNAc2Man5GlcNAc2Fuc1	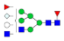	4%
	3228.19	NeuAc2Gal1GlcNAc1Man5GlcNAc2Fuc1	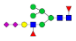	4%
	3646.33	NeuAc3Fuc1Gal2GlcNAc2Man3GlcNAc2Fuc1	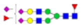	4%
	4072.63	GalNAc1NeuGc3Gal2GlcNAc3Man3GlcNAc2Fuc1	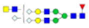	3%
	4287.6	NeuGc3Gal3GlcNAc4Man3GlcNAc2Fuc1	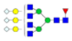	3%
	4461.8	Gal6GlcNAc7Man3GlcNAc2Fuc1	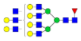	3%
Lubricating	1219.92	$Man_3GlcNAc_2$ + 1 Me	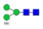	32%
	1430.07	$GlcNAc_1Man_2GlcNAc_2Xyl_1$	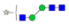	64%
	1568	$Man_3GlcNAc_2Fuc_1Xyl_1$	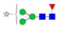	4%
Protective	1219.92	$Man_3GlcNAc_2$ + 1 Me	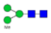	12%
	1400.13	$Man_3GlcNAc_2Fuc_1$	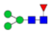	29%
	1430.07	$GlcNAc_1Man_2GlcNAc_2Xyl_1$	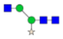	50%
	1796.24	$Man_4GlcNAc_2Fuc_2$	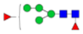	3%
	1853.33	$NeuAc_1Gal_1GlcNAc_1Man_2GlcNAc_2$	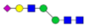	3%
	2067.43	$NeuAc_1Gal_1GlcNAc_1Man_3GlcNAc_2$	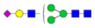	2%
	2280.56	$NeuAc_1Gal_1Hex_1GlcNAc_1Man_3GlcNAc_2$	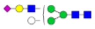	1%

Supplementary Table 11. Glycans extracted from adhesive *C. aspersum* snail mucus proteins that were detected via glycoproteomic tandem mass spectrometry analysis.

Mucus	Glycosylation Type	*m/z*	Glycan
Adhesive	O	203.07937	HexNAc(1)
Adhesive	O	365.1322	HexNAc(1)Hex(1)
Adhesive	O	406.15875	HexNAc(2)
Adhesive	O	527.18502	HexNAc(1)Hex(2)
Adhesive	O	541.20072	(Me)HexNAc(1)Hex(2)
Adhesive	O	568.21157	HexNAc(2)Hex(1)
Adhesive	O	656.22761	HexNAc(1)Hex(1)NeuAc(1)
Adhesive	O	673.24293	HexNAc(1)Hex(2)Fuc(1)
Adhesive	O	703.25354	(Me)HexNAc(1)Hex(3)
Adhesive	N	203.07937	HexNAc(1)
Adhesive	N	349.13728	HexNAc(1)Fuc(1)
Adhesive	N	406.15875	HexNAc(2)
Adhesive	N	495.19519	HexNAc(1)Fuc(2)
Adhesive	N	552.21665	HexNAc(2)Fuc(1)
Adhesive	N	568.21157	HexNAc(2)Hex(1)
Adhesive	N	698.27456	HexNAc(2)Fuc(2)
Adhesive	N	714.26948	HexNAc(2)Hex(1)Fuc(1)
Adhesive	N	730.26439	HexNAc(2)Hex(2)
Adhesive	N	860.32739	HexNAc(2)Hex(1)Fuc(2)
Adhesive	N	876.3223	HexNAc(2)Hex(2)Fuc(1)
Adhesive	N	892.31722	HexNAc(2)Hex(3)
Adhesive	N	906.33292	(Me)HexNAc(2)Hex(3)
Adhesive	N	1022.38021	HexNAc(2)Hex(2)Fuc(2)
Adhesive	N	1024.35947	HexNAc(2)Hex(3)Pent(1)
Adhesive	N	1038.37512	HexNAc(2)Hex(3)Fuc(1)
Adhesive	N	1052.39087	(Me2)HexNAc(2)Hex(3)Pent(1)
Adhesive	N	1054.37004	HexNAc(2)Hex(4)
Adhesive	N	1065.38602	HexNAc(3)Hex(2)Pent(1)
Adhesive	N	1095.39659	HexNAc(3)Hex(3)
Adhesive	N	1170.41738	HexNAc(2)Hex(3)Fuc(1)Pent(1)
Adhesive	N	1184.43303	HexNAc(2)Hex(3)Fuc(2)
Adhesive	N	1227.43885	HexNAc(3)Hex(3)Pent(1)
Adhesive	N	1241.4545	HexNAc(3)Hex(3)Fuc(1)
Adhesive	N	1257.44941	HexNAc(3)Hex(4)
Adhesive	N	1298.47596	HexNAc(4)Hex(3)
Adhesive	N	1346.48586	HexNAc(2)Hex(4)Fuc(2)
Adhesive	N	1362.48077	HexNAc(2)Hex(5)Fuc(1)
Adhesive	N	1378.47569	HexNAc(2)Hex(6)
Adhesive	N	1387.51241	HexNAc(3)Hex(3)Fuc(2)
Adhesive	N	1403.50732	HexNAc(3)Hex(4)Fuc(1)
Adhesive	N	1430.51822	HexNAc(4)Hex(3)Pent(1)
Adhesive	N	1519.55466	HexNAc(3)Hex(3)Fuc(2)Pent(1)
Adhesive	N	1540.52851	HexNAc(2)Hex(7)
Adhesive	N	1565.56014	HexNAc(3)Hex(5)Fuc(1)
Adhesive	N	1606.58669	HexNAc(4)Hex(4)Fuc(1)
Adhesive	N	1670.5915	HexNAc(2)Hex(6)Fuc(2)
Adhesive	N	1702.58133	HexNAc(2)Hex(8)
Adhesive	N	1752.6446	HexNAc(4)Hex(4)Fuc(2)
Adhesive	N	1850.69261	HexNAc(6)Hex(3)Fuc(1)
Adhesive	N	1900.68178	HexNAc(4)Hex(5)Fuc(1)Pent(1)
Adhesive	N	1914.69743	HexNAc(4)Hex(5)Fuc(2)
Adhesive	N	2336.85109	HexNAc(6)Hex(6)Fuc(1)
Adhesive	N	3432.24767	HexNAc(9)Hex(9)Fuc(1)

Supplementary Table 12. Glycans extracted from lubricating *C. aspersum* snail mucus proteins that were detected via glycoproteomic tandem mass spectrometry analysis.

Mucus	Glycosylation Type	*m/z*	Glycan
Lubricating	O	203.07937	HexNAc(1)
Lubricating	O	365.1322	HexNAc(1)Hex(1)
Lubricating	O	406.15875	HexNAc(2)
Lubricating	O	527.18502	HexNAc(1)Hex(2)
Lubricating	O	541.20072	(Me)HexNAc(1)Hex(2)
Lubricating	O	555.21642	(Me2)HexNAc(1)Hex(2)
Lubricating	O	568.21157	HexNAc(2)Hex(1)
Lubricating	O	673.24293	HexNAc(1)Hex(2)Fuc(1)
Lubricating	O	703.25354	(Me)HexNAc(1)Hex(3)
Lubricating	N	349.13728	HexNAc(1)Fuc(1)
Lubricating	N	406.15875	HexNAc(2)
Lubricating	N	495.19519	HexNAc(1)Fuc(2)
Lubricating	N	552.21665	HexNAc(2)Fuc(1)
Lubricating	N	568.21157	HexNAc(2)Hex(1)
Lubricating	N	698.27456	HexNAc(2)Fuc(2)
Lubricating	N	714.26948	HexNAc(2)Hex(1)Fuc(1)
Lubricating	N	730.26439	HexNAc(2)Hex(2)
Lubricating	N	860.32739	HexNAc(2)Hex(1)Fuc(2)
Lubricating	N	876.3223	HexNAc(2)Hex(2)Fuc(1)
Lubricating	N	892.31722	HexNAc(2)Hex(3)
Lubricating	N	906.33292	(Me)HexNAc(2)Hex(3)
Lubricating	N	1022.38021	HexNAc(2)Hex(2)Fuc(2)
Lubricating	N	1024.35947	HexNAc(2)Hex(3)Pent(1)
Lubricating	N	1038.37512	HexNAc(2)Hex(3)Fuc(1)
Lubricating	N	1052.39087	(Me2)HexNAc(2)Hex(3)Pent(1)
Lubricating	N	1054.37004	HexNAc(2)Hex(4)
Lubricating	N	1065.38602	HexNAc(3)Hex(2)Pent(1)
Lubricating	N	1095.39659	HexNAc(3)Hex(3)
Lubricating	N	1170.41738	HexNAc(2)Hex(3)Fuc(1)Pent(1)
Lubricating	N	1184.43303	HexNAc(2)Hex(3)Fuc(2)
Lubricating	N	1200.42795	HexNAc(2)Hex(4)Fuc(1)
Lubricating	N	1216.42286	HexNAc(2)Hex(5)
Lubricating	N	1227.43885	HexNAc(3)Hex(3)Pent(1)
Lubricating	N	1241.4545	HexNAc(3)Hex(3)Fuc(1)
Lubricating	N	1257.44941	HexNAc(3)Hex(4)
Lubricating	N	1298.47596	HexNAc(4)Hex(3)
Lubricating	N	1362.48077	HexNAc(2)Hex(5)Fuc(1)
Lubricating	N	1373.49676	HexNAc(3)Hex(3)Fuc(1)Pent(1)
Lubricating	N	1378.47569	HexNAc(2)Hex(6)
Lubricating	N	1387.51241	HexNAc(3)Hex(3)Fuc(2)
Lubricating	N	1403.50732	HexNAc(3)Hex(4)Fuc(1)
Lubricating	N	1524.5336	HexNAc(2)Hex(6)Fuc(1)
Lubricating	N	1535.54958	HexNAc(3)Hex(4)Fuc(1)Pent(1)
Lubricating	N	1540.52851	HexNAc(2)Hex(7)
Lubricating	N	1565.56014	HexNAc(3)Hex(5)Fuc(1)
Lubricating	N	1702.58133	HexNAc(2)Hex(8)
Lubricating	N	1864.63416	HexNAc(2)Hex(9)
Lubricating	N	1868.69195	HexNAc(4)Hex(3)Fuc(3)Pent(1)
Lubricating	N	1884.68686	HexNAc(4)Hex(4)Fuc(2)Pent(1)
Lubricating	N	2046.73968	HexNAc(4)Hex(5)Fuc(2)Pent(1)
Lubricating	N	2188.7398	HexNAc(2)Hex(11)
Lubricating	N	2192.79759	HexNAc(4)Hex(5)Fuc(3)Pent(1)
Lubricating	N	2336.85109	HexNAc(6)Hex(6)Fuc(1)

Supplementary Table 13. Glycans extracted from protective *C. aspersum* snail mucus proteins that were detected via glycoproteomic tandem mass spectrometry analysis.

Mucus	Glycosylation Type	*m/z*	Glycan
Protective	O	203.07937	HexNAc(1)
Protective	O	365.1322	HexNAc(1)Hex(1)
Protective	O	406.15875	HexNAc(2)
Protective	O	527.185	HexNAc(1)Hex(2)
Protective	O	541.20072	(Me)HexNAc(1)Hex(2)
Protective	O	555.21642	(Me2)HexNAc(1)Hex(2)
Protective	O	568.2115	HexNAc(2)Hex(1)
Protective	O	656.22761	HexNAc(1)Hex(1)NeuAc(1)
Protective	O	673.24293	HexNAc(1)Hex(2)Fuc(1)
Protective	O	703.25354	(Me)HexNAc(1)Hex(3)
Protective	O	859.30699	HexNAc(2)Hex(1)NeuAc(1)
Protective	O	947.32303	HexNAc(1)Hex(1)NeuAc(2)
Protective	O	1021.35981	HexNAc(2)Hex(2)NeuAc(1)
Protective	O	1151.4228	HexNAc(2)Hex(1)Fuc(2)NeuAc(1)
Protective	O	1238.41845	HexNAc(1)Hex(1)NeuAc(3)
Protective	O	1312.45523	HexNAc(2)Hex(2)NeuAc(2)
Protective	N	203.07937	HexNAc(1)
Protective	N	349.13728	HexNAc(1)Fuc(1)
Protective	N	406.15875	HexNAc(2)
Protective	N	495.19519	HexNAc(1)Fuc(2)
Protective	N	552.21665	HexNAc(2)Fuc(1)
Protective	N	568.21157	HexNAc(2)Hex(1)
Protective	N	698.27456	HexNAc(2)Fuc(2)
Protective	N	714.26948	HexNAc(2)Hex(1)Fuc(1)
Protective	N	730.26439	HexNAc(2)Hex(2)
Protective	N	860.32739	HexNAc(2)Hex(1)Fuc(2)
Protective	N	876.3223	HexNAc(2)Hex(2)Fuc(1)
Protective	N	892.31722	HexNAc(2)Hex(3)
Protective	N	906.33292	(Me)HexNAc(2)Hex(3)
Protective	N	1022.38021	HexNAc(2)Hex(2)Fuc(2)
Protective	N	1024.35947	HexNAc(2)Hex(3)Pent(1)
Protective	N	1052.39087	(Me2)HexNAc(2)Hex(3)Pent(1)
Protective	N	1065.38602	HexNAc(3)Hex(2)Pent(1)
Protective	N	1095.39659	HexNAc(3)Hex(3)
Protective	N	1184.43303	HexNAc(2)Hex(3)Fuc(2)
Protective	N	1216.42286	HexNAc(2)Hex(5)
Protective	N	1241.4545	HexNAc(3)Hex(3)Fuc(1)
Protective	N	1257.44941	HexNAc(3)Hex(4)
Protective	N	1298.47596	HexNAc(4)Hex(3)
Protective	N	1362.48077	HexNAc(2)Hex(5)Fuc(1)
Protective	N	1373.49676	HexNAc(3)Hex(3)Fuc(1)Pent(1)
Protective	N	1378.47569	HexNAc(2)Hex(6)
Protective	N	1419.50224	HexNAc(3)Hex(5)
Protective	N	1430.51822	HexNAc(4)Hex(3)Pent(1)
Protective	N	1444.53387	HexNAc(4)Hex(3)Fuc(1)
Protective	N	1508.53868	HexNAc(2)Hex(5)Fuc(2)
Protective	N	1524.5336	HexNAc(2)Hex(6)Fuc(1)
Protective	N	1535.54958	HexNAc(3)Hex(4)Fuc(1)Pent(1)
Protective	N	1540.52851	HexNAc(2)Hex(7)
Protective	N	1549.56523	HexNAc(3)Hex(4)Fuc(2)
Protective	N	1565.56014	HexNAc(3)Hex(5)Fuc(1)
Protective	N	1702.58133	HexNAc(2)Hex(8)
Protective	N	1864.63416	HexNAc(2)Hex(9)
Protective	N	1868.69195	HexNAc(4)Hex(3)Fuc(3)Pent(1)
Protective	N	1884.68686	HexNAc(4)Hex(4)Fuc(2)Pent(1)
Protective	N	1914.69743	HexNAc(4)Hex(5)Fuc(2)
Protective	N	2012.74544	HexNAc(6)Hex(4)Fuc(1)
Protective	N	2026.68698	HexNAc(2)Hex(10)
Protective	N	2046.73968	HexNAc(4)Hex(5)Fuc(2)Pent(1)
Protective	N	2188.7398	HexNAc(2)Hex(11)
Protective	N	2192.79759	HexNAc(4)Hex(5)Fuc(3)Pent(1)
Protective	N	3432.24767	HexNAc(9)Hex(9)Fuc(1)

Supplementary Methods

1. Mucus collection

In October 2021, snail mucus was collected directly from *C. aspersum* snails (provided by Peconic Escargot in Cutchogue, NY) in three separate manners to differentiate by function, as described previously.[1,2] Snails were cultured at room temperature and provided a diet of dirt, wild herbs, and cultivated herbs *ad libitum*. 25 physically active snails that were between 5 – 7 cm were washed with room temperature tap water to remove food, debris, and pathogens and placed into a plastic aquarium. Lubricating mucus was collected by allowing snails to crawl along horizontal petri dishes to deposit the secretion used to facilitate movement. Adhesive mucus was collected by holding snails against inverted petri dishes to induce adhesion, and the snails were left suspended for 15 min such that they deposited onto the dish the mucus used in adhesion. Lubricating and adhesive mucus were not processed or manipulated further and were immediately place on ice for preservation. Protective mucus was collected by gently scraping the dorsal surface of the snail with a spatula, collecting the skin mucus and depositing it in a collection tube. Samples were stored under ice packs without furhter processing in an insulated cooler for transport and then stored in an ultralow temperature (–80 ˚C) freezer until use.

2. Mucus purification.

Mucus samples were thawed and physical debris was removed with tweezers. 2 mL of 6 M Guanidinium HCl (Gdn), CsCl (density 1.388 g/mL, measured by gravimetric analysis) was added to mucus-containing petri dishes and collection tubes and incubated at 4 ^{o}C overnight on an Ohaus RockingShaker orbital shaker to dissolve mucus, as described previously.[3] After overnight incubation, the mucus-containing solutions in the petri dishes were pooled by mucus type into 13.2 mL ultracentrifuge tubes (Beckman-Coulter). Additional Gdn solution and mucus residue were collected by gently scraping the petri dishes with a razor blade. Samples were then subjected to isopycnic density gradient ultracentrifugation in a swinging bucket SW41 Ti rotor in a Beckman-Coulter Optima XE Ultracentrifuge (35,000 rpm, 72 hr, 4 ^{o}C), at a relative centrifugal force of 150,000 x g, within which mucus migrates to a characteristic band and cells would be removed from the solution, as described previously.[4] Following centrifugation, tubes were pierced with a needle and fractionated (0.5 – 1 mL fractions). Notably, the mucus samples were colorless aggregates about two-thirds up the gradient. Additionally, each fraction was measured for density and tested for carbohydrate content using a microtiter periodic acid-Schiff's reagent (PAS) staining protocol, as described previously.[5] 25 μL of each fraction was added to each well of a clear, flat-bottom 96 well plate. 120 μL of 0.06 % w/v perioidic acid, 7 % v/v glacial acetic acid in water, was added to each well in the dark and covered in tin foil and left to incubate for 90 min at 25 ˚C. After incubation, 100 μL of Schiff's reagent was added to each well in the dark and covered to incubate for 60 min at 25 ˚C. The plate was then subjected to spectrophotometric analysis, and absorbance was measured at 550 nm using a Molecular Devices SpectraMAX 190 microplate reader. Additionally, the densities of each fraction were determined by gravimetric analysis, measuring the mass of 500 μL of each fraction. Fractions with a density of approximately 1.4 g/mL as well as high signal-to-background absorbance at 550 nm were considered mucin-positive because it has been reported mucins exhibit a characteristic buoyancy, migrating to this fraction of the density gradient, and glycans labelled with Schiff's stain exhibit an absorbance maximum at 550 nm.[4] Mucin-positive fractions were pooled and dithiothreitol (DTT) was added to each pool to reach a final concentration of 0.05 M DTT, and shaken at 45 ^{o}C overnight in an Echotherm orbital mixing dry bath (Torrey Pines Scientific) to reduce disulfide bonds in the mucus hydrogel networks. Reduced samples were then dialyzed in a cellulose membrane (MM cutoff 2 kDa) against 3 changes of ultrapure water over 48 h and flocculent beige precipitate formed. Samples were then lyophilized using a Labconco Freezedry-System / Freezone 4.5 at –55 ^{o}C / 1 mbar, resulting in a light beige powder which was stored at –80 ^{o}C. Protein content in each collected mucus sample was quantified at each step in the purification using the Nanodrop one-C spectrophotometer (Thermo-Fisher), comparing values to protein concentration standard curves. Standard curves of absorbance vs. protein concentration (in mg/mL) were generated using the same Nanodrop one-C spectrophotometer, and tracking the absorbance signal at 280 nm for varying concentrations of protein. Solutions for the standard curve were generated by dissolving dried mucus protein in 6 M Gdn solution and mixing.

3. Proteomic analysis

RNA Extraction and Sequencing

Snails provided by Peconic Escargot in February 2020 were sacrificed on-site via freezing in a dry ice-ethanol mixture. Whole snails were stored in Invitrogen RNAlater™ (Thermo Fisher, AM7021) and frozen at –80 °C until used. 6 individual tissue slices of the snail's dorsal and pedal surfaces of the foot were excised from different snails. Total RNA was extracted from these slices using a Qiagen RNeasy Micro kit (Qiagen, 74004) according to manufacturer's instructions. The integrity of total RNA was confirmed using nanodrop and Agilent 2100 BioAnalyzer analysis. The RNA Integrity Number (RIN) was not considered because of known co-migration of 28S rRNA fragments with 18S rRNA in molluscan RNA.[6,7] Total RNA was used as a template to perform polyA enriched first strand cDNA synthesis using the HiSeq RNA sample preparation kit for Illumina Sequencing (Illumina Inc., CA) following manufacturer's instructions. The cDNA libraries were sequenced using Illumina HiSeq 1000 technology using a paired end flow cell and 80 x 2 cycle sequencing.

Read Processing and De Novo Assembly

Raw reads were quality checked with FastQC v0.11.5 (www.bioinformatics.babraham.ac.uk).[8] Adapter sequences and low-quality reads (Phred score <33) were removed using Trimmomatic v0.36 and trimmed reads were re-evaluated with FastQC to ensure the high quality of the data after the trimming process.[9] Due to the lack of a reference genome, the processed reads were de novo assembled using Trinity v2.4.0.[10] De novo assembled transcriptomes were translated with Trinity Super Transcripts.[11] Supertranscripts was used to construct the largest isoform of each gene, in other words producing the original unspliced transcripts, rather than spliced variants of the transcripts.[11] 179,552 transcripts were assembled. RNA sequences were deposited in Genbank with the BioSample accession codes SAMN29856567, SAMN29856568, SAMN29856569, SAMN29856570, SAMN29856571, SAMN29856572, and SRA accession codes SRR20337023, SRR20337022, SRR20337021, SRR20337020, SRR20337019, SRR20337018..

SDS-PAGE

Lyophilized snail mucus protein samples were suspended in 25 µL of ultrapure water and concentrations were verified via Nanodrop, and then diluted with ultrapure water to a concentration of 20 mg/mL. Samples were then mixed with an equal volume of 2X SDS loading buffer (Quality Biological, 351-082-661) with 5% (v/v) 2-mercaptoethanol (VWR, M31) and 1 % (v/v) Tween added. Samples were vortexed and incubated at 95 °C for 15 min using a VWR Mini Block Heater (10153-318). 10 µL of reduced protein samples were loaded in triplicate, alongside a Chameleon Duo Pre-Stained protein ladder (LI-COR, 928-60000) onto a 15-well 4 – 20 % Tris-glycine precast gradient gel (BioRad, 4561096) in a BioRad Mini-Protean Tetra system with PowerPac Basic. Gels were electrophoresed at 150 V for 54 min. Following electrophoresis, gels were rinsed with deionized water and each gel was divided into 3 pieces for Coomassie, silver, and PAS staining, respectively. From each gel, a control band from lanes that did not contain protein was sliced to verify gels were not contaminated.

For Coomassie protein staining, gels were fixed in 50 % (v/v) methanol, 10 % (v/v) acetic acid in water solution for 15 min. Gels were then stained in the same solution containing 1% (w/v) Coomassie Brilliant Blue G250 using microwave irradiation. Gels were destained by alternating washes with 50% methanol, 10% acetic acid solution, and ultrapure water.

For silver staining, gels were stained using the Pierce Silver Staining for Mass Spectrometry kit (Thermo Scientific, 24600) according to manufacturer's instructions.

For PAS staining, gels were incubated in 1 % (w/v) periodic acid solution in the dark for 5 min with occasional shaking. Gels were rinsed with water and incubated in 0.5 % (w/v) sodium metabisulfite solution for 5 min. Gels were rinsed with water and incubated in Schiff's reagent for 5 min in the dark with occasional shaking. Gels were rinsed with water and incubated in 0.5 % (w/v) sodium metabisulfite solution for 5 min. Gels were destained by alternating washes with 50% methanol, 10% acetic acid solution and ultrapure water.

All gels were imaged simultaneously on an Aversham Imager 600 gel imager (GE) using colorimetric transillumination. Following imaging, Coomassie-stained gel slices were excised using a scalpel. Slices were excised to obtain molecular mass ranges of < 40 kDA, 40 – 150 kDA, and 150+ kDa. Gel slices were stored on ice packs for transport prior to proteomic analysis.

Proteomic Mass Spectrometry of Purified Mucus Proteins

Purified snail mucus protein samples were loaded onto a single stacking gel band to remove lipids, detergents and salts. The single gel band containing all proteins was reduced with dithiothreitol (DTT), alkylated with iodoacetic acid and digested with trypsin. 2 μg of extracted peptides were re-solubilized in 0.1 % aqueous formic acid and loaded onto a Thermo Acclaim Pepmap (Thermo, 75 μM ID X 2 cm C18 3 μM beads) precolumn and then onto an Acclaim Pepmap Easyspray (Thermo, 75 μM X 15 cm with 2 μM C18 beads) analytical column separation using a Dionex Ultimate 3000 uHPLC at 250 nL/min with a gradient of 2-35 % organic (0.1 % formic acid in acetonitrile) over 1 hr. Peptides were analyzed using a Thermo Orbitrap Fusion mass spectrometer operating at 120,000 resolution (FWHM in MS1) with HCD sequencing (15,000 resolution) at top speed for all peptides with a charge of 2+ or greater.

Proteomic Mass Spectrometry of SDS-PAGE Gel Bands

Gel bands were reduced with DTT, alkylated with iodoacetic acid and digested with trypsin. Extracted peptides were re-solubilized in 0.1% aqueous formic acid and loaded onto a Thermo Acclaim Pepmap (Thermo, 75uM ID X 2cm C18 3uM beads) precolumn and then onto an Acclaim Pepmap Easyspray (Thermo, 75uM X 15cm with 2uM C18 beads) analytical column. Separation was conducted using a Dionex Ultimate 3000 uHPLC at 250 nl/min with a gradient of 2-35% organic (0.1% formic acid in acetonitrile) over 2 hours. Peptides were analyzed using a Thermo Orbitrap Fusion mass spectrometer operating at 120,000 resolution (Full Width at Half Maximum in MS1) with HCD sequencing (15,000 resolution) at top speed for all peptides with a charge of 2+ or greater.

Proteomic Data Processing

The raw data were converted into *.mgf format (Mascot generic format) for searching using the Mascot 2.6.2 search engine (Matrix Science) against predicted sequences from the *de novo* assembled snail transcriptome.[12] The database search results were loaded onto Scaffold Q+ Scaffold_4.9.0 (Proteome Sciences) for statistical treatment and data visualization.[13] Peptide identifications were made by exact homology of fragmented peptides against translated transcripts. Using the Scaffold Local FDR (false discovery rate) algorithm, probability thresholds for peptide identifications and protein identifications were set at 95.0 % and 5.0 %, respectively, to achieve an FDR less than 1.0 %, as per proteomic research standards.[14-16] Additionally, accepted sequences

must have contained at least 2 identified peptides. Peptides were quantified by MS/MS counts. The mass spectrometry proteomics data have been deposited to the ProteomeXchange Consortium via the PRIDE partner repository with the dataset identifier PXD035534 and 10.6019/PXD035534.[17]

Bioinformatic Analysis

The sequences of the proteins identified in the mucus samples were subjected to BLASTP searches using default parameters to determine their functions based on homology with known proteins in the NCBI non-redundant protein database.[18] Each protein was manually classified into one of nine functional categories: lectin, glycoprotein, network-formation, matrix, enzymes, protease inhibitors, ion-binding, regulatory, or housekeeping. Proteins that had similarity with predicted snail proteins without known function were classified as “Snail,” and proteins that had no similarity with any known proteins were classified as “Novel.” Sequences were uploaded into ClustalW to generate a dendrogram.[19] Molluscan proteins of each functional category, as well as three human mucins, were included in the tree generation. Protein sequences from *Amphioctopus fangsiao, Aplysia californica, Argopecten irradians, Biomphalaria glabrata, Bulinus truncates, Cernuella virgata, Cornu aspersum, Crassostrea gigas, Crassostrea hongkongensis, Crassostrea virginica, Elysia marginata, Gigantopelta aegis, Haliotis discus, Haliotis rubra, Haliotis tuberculata, Helix pomatia, Hemitoma cumingii, Homo sapiens, Mercenaria mercenaria, Meretrix meretrix, Mizuhopecten yessoensis, Mus musculus, Mytilus coruscus, Mytilus edulis, Mytilus galloprovincialis, Octopus sinensis, Onchidium reevesii, Patella vulgata, Pectin Maximus, Pinctada fucata, Plakobranchus ocellatus, Pomacea canaliculata, Sepia pharaonis,* and *Vampyroteuthis infernalis,* were used to generate the dendrogram. Three proteins from *Mus musculus*, (Pikachurin1, Pikachurin2, and Pikachurin3), were included as an outgroup. Display and annotation of dendrogram was conducted using iTOL v5.[20] Sequences were uploaded into the HMMER web server for identification of domains.[21] Multiple sequence alignment of proteins was conducted using Jalview v2.11.2.7.[22]

Glycoproteomic Tandem Mass Spectrometry:

Purified mucus protein samples (n = 3, 3 biological replicates) were resuspended in 100 µL of 50 mM ammonium bicarbonate. To this, 100 µL of 25 mM DTT was added. Samples were vortexed and incubated at 45°C for 45 minutes. Following incubation, samples were allowed to cool to room temperature, and 100 µL of 90 mM iodoacetamide (IAA) was added. Samples were vortexed and incubated at room temperature in the dark for 20 minutes. Following incubation, samples were cleaned with 3 kDa MWCO filters (Millipore Amicon Ultra, UFC500396). Prior to loading samples, the filters were washed twice with 400 µL of 50 mM ammonium bicarbonate. Filters were spun for 10 minutes at 14000 rpm. Following washing, samples were loaded onto the filters and spun at 14000 rpm for 25 minutes. 400 µL of 50 mM ammonium bicarbonate was added, and samples were spun once more. This process was repeated one more time. The desalted protein sample was removed from the filter by inverting it into a clean tube and centrifuging for 5 minutes. The filters were rinsed with 50 µL of 50 mM ammonium bicarbonate, and the samples were inverted once more and centrifuged. The total volume was then brought to 100 µL. A 5 µL aliquot was digested with 1 µg of trypsin (Promega) overnight at 37°C. Trypsin was then terminated by heating samples to 100°C for 5 minutes. Samples were then passed through a 0.2 µm filter. Samples were diluted to 30 µL in 0.1% formic acid.

Samples were analyzed using a Thermo Fisher Eclipse Tribrid mass spectrometer equipped with a nano electrospray source and coupled to an Ultimate 3000 RSLCnano liquid chromatography system. Samples were analyzed using a 180-minute gradient. A prepacked nano-LC column of 15 cm length and 75 µm internal diameter, filled with 3 µm C18 material was used. The precursor ion scan was acquired at 120,000 resolution in the Orbitrap, and precursors with a time frame of 3 seconds were selected for MS/MS fragmentation in the Orbitrap at 15,000 resolution. Monoisotopic precursor selection was selected and the threshold for MS/MS triggering was 1000 counts. MS/MS fragmentation was done using stepped higher energy collision induced dissociation (HCD) product triggered collision induced dissociation (CID) (HCDpdCID). Precursors with an unknown charge state or charge state of +1 were excluded, and samples were run in positive ion mode. [23]

LC-MS/MS spectra were searched against the FASTA sequences of the Helicidae genome obtained from Uniprot, as well as the protein sequences obtained from proteomics experiments and RNAseq experiments outlined in this paper. Byonic software (Ver 5.0) was used for data analysis. Oxidation of methionine, deamidation of asparagine and glutamine were searched as variable modifications and carbamidomethylation of cystine was searched as a fixed modification. A Byonic N-glycan database of insect and plant glycans as well as a tailored glycan list developed based on the glycomics results were searched as variable modification for *N*-glycans. A Byonic *O*-glycan database of 9 common *O*-glycans as well as a tailored glycan list developed based on our glycomics results were searched as variable modification for *O*-glycans. The data was then filtered based on a |log prob| value equal to or greater than 3, and a Delta Mod Score equal to or greater than 50.[24] Matches were manually verified by confirming presence of oxonium ions and expected neutral loss patterns. To identify glycoprotein candidates attributed to prominent 260 kDa bands in all three samples, peptides were extracted and analyzed by LC-MS/MS on the same instrument for glycoproteomic characterization. Entire duty cycle was used for stepped HCD fragmentation for high confidence peptide backbone sequencing. Common contaminants and decoys generated by Byonic software were added to the FASTA databases to interrogate candidate components in the bands. Glycoproteomics data were submitted to GlycoPost database under the accession number GPST000297.

4. Glycomic analysis

Reduction and N-glycan Release

Following purification, the lyophilized samples were resuspended in 25 µL of 50 mM NH_4HCO_3 buffer. To this, 25 µL of 25 mM dithiothreitol (DTT) was added and the samples were vortexed. The samples were then incubated at 50°C for 45 minutes. Following incubation, the samples were allowed to come to room temperature. Samples were then cleaned and desalted using Amicon Ultra 10 kDa molecular weight cut off (MWCO) filters (Millipore). The filters were first filled with water (500 µL) and centrifuged at 14 000 x g for 10 mins. Resultant flow through was discarded. Then, the sample mixture was loaded onto the filter, and the filter was again centrifuged for 10 min at 14 000 x g. 500 µL of 50 mM NH_4HCO_3 was then loaded onto the filter and centrifuged one more time at 14 000 x g for 10 minutes. The flow through was discarded, and the desalted mucus protein sample remained in the filter. To remove the sample from the filter, the filter was inverted into a clean tube and centrifuged for 1 minute. The filter was then rinsed with 20 µL of 50 mM NH_4HCO_3, inverted into the tube containing the sample, and again centrifuged for 1 minute. To this, 2 µL of PNGase F (New England Biolabs) was added. The samples were briefly vortexed and then incubated at 37°C for 48 hours.

Following incubation with PNGase F, the samples were once again passed through a 10 kDa MWCO filter using the conditions stated above. Following centrifugation, 500 µL of 50 mM ammonium bicarbonate was added and centrifuged once more. The flow through, which contained the released *N*-glycans, was then loaded onto a C18 SPE cartridge (Resprep). The C18 cartridge was first washed with 1 mL of methanol (MeOH) and conditioned with 3 mL of 5% acetic acid. The samples were then loaded onto the C18 column and allowed to flow through. N-glycans were then eluted from the column with 3 mL of 5% acetic acid, and the resultant flow through was lyophilized. The de-N-glycosylated protein sample, which remains in the filter, was removed from the filter as stated above. The de-*N*-glycosylated samples were then lyophilized.

β-elimination and O-glycan Release

Following lyophilization, the de-*N*-glycosylated protein was subjected to *β*-elimination. The samples were dissolved in 250 µL of 100 mM NaOH solution and vortexed. The pH of the sample was then checked using pH paper to ensure basic conditions (pH ranged from 10-13). Then, 55 mg/mL of $NaBH_4$ in 100 mM NaOH was added and the sample was vortexed. The sample was then incubated at 50°C for 52 hours. Following incubation samples were neutralized by adding 10% acetic acid dropwise, and vortexed between each addition. As the acid is added the sample bubbles, and this process was repeated until bubbling ceased.

Poly-prep chromatography columns (Bio-Rad) were packed with H^+ activated ion exchange resins (DOWEX™ 50W x 8-100) and rinsed 5 times with 1 mL of 5% acetic acid. Following rinsing, the neutralized samples were loaded onto the DOWEX columns and allowed to flow through. The flow through was then loaded onto a C18 SPE column, which was washed and conditioned as described above. 3mL of 5% acetic acid was then loaded onto the DOWEX column to elute the oligosaccharides, and this flow through was once again loaded onto the C18 SPE cartridge. Samples were then frozen on dry ice and lyophilized.

Following lyophilization the borates were removed using 9:1 MeOH: acetic acid. 500 µL of the mixture was added to the samples, vortexed and dried under a stream of N_2 gas. This process was repeated until the borates were fully removed (approximately 5 times).

Per-O-methylation of N- and O-linked Glycans

The *N*- and *O*-linked glycans were then per-*O*-methylated using NaOH/dimethyl sulfoxide (DMSO) base and iodomethane. First, the NaOH/DMSO base was made by adding 100 µL of 50% (v/v) NaOH to a clean, dry glass vial. Then 200 µL of MeOH was added and the solution was then mixed. 4 mL of DMSO was then added and the vial was mixed vigorously for 3 minutes. The sample was then centrifuged at 3000 rcf for 5 mins. A white precipitate forms at the top of the vial, and a clear base at the bottom. The white precipitate and all remaining DMSO were removed without disturbing the base, and 4 mL more of DMSO was added. This procedure was repeated until white precipitate no longer forms (approximately 3 times). 1 mL of DMSO was then added and homogenized with the clear base.

The samples were dissolved in 200 µL of DMSO and vortexed. Then, 300 µL of the NaOH/DMSO base was added, and to this, 100 µL of iodomethane, or iodomethane-D3 was added and the sample was mixed vigorously for 20 minutes using a shaker.[25] The reaction was then quenched using 2 mL of LC-MS grade water. Turbidity was and should be observed. The iodomethane was then removed by bubbling N_2 into the sample for approximately 3 minutes. Then, 2 mL of dichloromethane was added, and the sample was mixed vigorously. The sample was then centrifuged at 3000 rcf for 1 minute for phase separation. The upper water layer was then removed, and to additional mL of water was added. This process was repeated a total of five times. Following the last wash, all traces of water was removed, and the dichloromethane fraction was transferred to a clean glass vial. The sample was then dried under a stream of N_2. The sample was then resuspended in 20 µL of MeOH and analyzed using MALDI-TOF-MS.

Mass Spectrometry of Per-O-methylated glycans

2 µL of the per-*O*-methylated samples (n = 3, 3 biological replicates) were mixed with 2 µL of 2,5-dihydroxybenzoic acid (DHB) MALDI matrix. DHB was made at a concentration of 15 mg/mL in 70:30:0.1 acetonitrile: water: formic acid. 1 µL of the mixture was spotted on a MALDI plate and allowed to dry. The MALDI plate (stainless steel) was then analyzed using an AB Sciex TOF/TOF 5800 System Mass Spectrometer in positive ion mode using reflector mode. This system contains a nitrogen laser (337 nm wavelength) and the laser intensity used was 6400 (arbitrary units). Only MS1 data was collected, no MS/MS or in-source decay was performed. 2500 shots per spectrum were collected. Fetuin was processed identically as a glycoprotein control.

Quantification of Glycans using Internal Standard

To determine the amount of glycan in the samples and internal standard of a known concentration was added. Xylotetraose (Megazyme) was permethylated separately using the procedure outlined above. 0.1 µg of the xylose standard was added to the oligosaccharide-DHB mixture and spotted on a MALDI plate. Concentrations of the glycans released from the mucus protein samples were determined by comparing the peak intensities to that of the standard.

Assignment of Glycan Structures

Glycan compositions were determined using SCIEX Data Explorer software v2.2,[26] GlycoWorkbench 2.0 and manual interpretation.[27] Structural assignments were determined based on mass measurement, literature and biological probability. Glycomics data were submitted to GlycoPost database under the accession number GPST000297.

5. Scanning electron microscopy

The samples were made by letting the snail to crawl and leave mucus on an aluminum SEM pin stubs, similarly to the silicon wafer samples for AFM, and air-dried overnight. The samples were sputter-coated with gold to a thickness of 5 nm using a Leica EM ACE600 Coater for better electrical conductivity. These samples were then imaged in a Thermo Scientific (FEI) Helios NanoLab 660 FIB-SEM with HT of 5 kV, current of 6.3, 13 and 25 pA with ETD (Everhart-Thornley) detector. EDS (energy-dispersive X-ray spectroscopy) mapping was collected with an Oxford detector at HT of 10 kV and current of 1.6 nA. Data was collected and analyzed using AZtec v6.0 software.[28]

6. Atomic force microscopy

AFM topography

The sample was made by letting the snail to crawl and leave mucus on a silicon wafer, which was subsequently scanned by using an AFM (Multimode 8, Bruker). Mucus topographies were imaged using the AFM tapping mode with a probe (SCANASYST-AIR, Bruker) that has a tip radius of 20 nm. To locate positions of interest, we used a microscope to find sample features that are clean and intact, made the AFM probe to be above the feature, and then lowered the AFM probe to enable tip-sample interaction that is required for topography measurement. We controlled the scan size to be 10-20 um depending on the feature sizes, scan rate to be 0.8 Hz, and the pixel number to be 256 x 256. Each sample was scanned for more than 5 topographies images to avoid site selection bias and to ensure sample characteristics are captured.

Nano-indentation experimental procedure

The stiffness of mucus samples was characterized using the AFM nano-indentation method,[29] where an indenter (MLCT-E, Bruker) with radius of 20 nm and a spring constant of 0.139 N/m was used. The indentation deflection sensitivity was 40.7 nm/V, calibrated by performing an indentation on the silicon wafer substrate. To select the points for indentations, we first used the AFM tapping mode to map the surface materials, which were subsequently grouped into 3 to 5 categories based on their size and shape. For example, aggregates having sharp edges and have the size of ~1 um are regarded as one type; the material that spans across the entire material map and supports all kinds of aggregates is regarded as the other type. After we identified the material types on the sample, we offset the scanning tip to the peak of certain material type and decreased the scan size to 10 nm x 10 nm before we switched the AFM mode from the tapping mode to the indentation mode. The indentation rate was set at 1 Hz and the tip deflection signal, which triggers the approaching movement to switch to the retracting movement, was tuned to increase from 0.05 V until the voltage that leads to an indentation depth of ~2 nm, of which the indentation profiles were collected three times for analysis (Methods). Three locations of each material type were characterized to obtain statistically reliable stiffness results.

Stiffness and work of adhesion characterization via the JKR model

The stiffness of mucus samples was characterized using AFM nano-indentation method,[29] where an indenter (MLCT-E, Bruker) with radius of 20 nm and a spring constant of 0.139 N/m was used (NanoScope Analysis 1.9). The indentation deflection sensitivity was 40.7 nm/V, calibrated by performing an indentation on the silicon wafer substrate. Peaks of three mucus aggregates are indented to obtain the force *vs.* displacement relationships, of which the retracting portion of the indenting profiles were subsequently analyzed by using the Johnson–Kendall–Roberts (JKR) model, given by

(1) $E_{\mathrm{JKR}} = \frac{9\pi \mathrm{R}^2 \Delta r}{2a_0^3}$,

(2) $P_{\mathrm{adh}} = -\frac{3}{2}\pi \Delta r \mathrm{R}$,

(3) $h_{\mathrm{t}} - h_0 = \frac{a_0^2}{\mathrm{R}}\left(\frac{1+\sqrt{1-\frac{P}{P_{\mathrm{adh}}}}}{2}\right)^{\frac{4}{3}} - \frac{2}{3}\frac{a_0^2}{\mathrm{R}}\left(\frac{1+\sqrt{1-\frac{P}{P_{\mathrm{adh}}}}}{2}\right)^{\frac{1}{3}}$,

where E_{JKR} is the Young's modulus, R is the tip radius, Δr is the work of adhesion, a_0 is the contact area when the contract force is zero, P_{adh} is the pull-off force, h_{t} is the indentation depth, h_0 is the contact point where the pull-off force shows, and P is the load. The work of adhesion was measured by the area enclosed by the approaching and the retracting indentation force-displacement curves, and was normalized by the probe sample contact area (a_0), given by

(4) $a_0 = \pi R h_{\mathrm{t}}$.

Force-retract curves were selected for analysis if R^2 values were greater than .96 and E and W had non-negative values.

Supplementary References

1 Newar, J. & Ghatak, A. Studies on the adhesive property of snail adhesive mucus. *Langmuir* **31**, 12155-12160 (2015).

2 Greistorfer, S. *et al.* Snail mucus− glandular origin and composition in *Helix pomatia*. *Zoology* **122**, 126-138 (2017).

3 Ballance, S. *et al.* Partial characterisation of high-molecular weight glycoconjugates in the trail mucus of the freshwater pond snail Lymnaea stagnalis. *Comparative Biochemistry and Physiology Part B: Biochemistry and Molecular Biology* **137**, 475-486 (2004).

4 Corfield, A. P. *Glycoprotein methods and protocols: The mucins*. Vol. 125 (Springer Science & Business Media, 2000).

5 Kilcoyne, M., Gerlach, J. Q., Farrell, M. P., Bhavanandan, V. P. & Joshi, L. Periodic acid–Schiff's reagent assay for carbohydrates in a microtiter plate format. *Analytical biochemistry* **416**, 18-26 (2011).

6 Dheilly, N. M. *et al.* A family of variable immunoglobulin and lectin domain containing molecules in the snail *Biomphalaria glabrata*. *Developmental & Comparative Immunology* **48**, 234-243, doi:https://doi.org/10.1016/j.dci.2014.10.009 (2015).

7 Barcia, R., Lopez-García, J. M. & Ramos-Martínez, J. I. The 28S fraction of rRNA in molluscs displays electrophoretic behaviour different from that of mammal cells. *IUBMB Life* **42**, 1089-1092 (1997).

8 Brown, J., Pirrung, M. & McCue, L. A. FQC Dashboard: integrates FastQC results into a web-based, interactive, and extensible FASTQ quality control tool. *Bioinformatics* **33**, 3137-3139 (2017).

9 Bolger, A. M., Lohse, M. & Usadel, B. Trimmomatic: a flexible trimmer for Illumina sequence data. *Bioinformatics* **30**, 2114-2120 (2014).

10 Haas, B. J. *et al.* De novo transcript sequence reconstruction from RNA-seq using the Trinity platform for reference generation and analysis. *Nature protocols* **8**, 1494-1512 (2013).

11 Davidson, N. M., Hawkins, A. D. & Oshlack, A. SuperTranscripts: a data driven reference for analysis and visualisation of transcriptomes. *Genome biology* **18**, 1-10 (2017).

12 Helsens, K., Martens, L., Vandekerckhove, J. & Gevaert, K. MascotDatfile: an open-source library to fully parse and analyse MASCOT MS/MS search results. *Proteomics* **7**, 364-366 (2007).

13 Searle, B. C. Scaffold: a bioinformatic tool for validating MS/MS-based proteomic studies. *Proteomics* **10**, 1265-1269 (2010).

14 Liu, W. *et al.* Stress-Induced Mucus Secretion and Its Composition by a Combination of Proteomics and Metabolomics of the Jellyfish *Aurelia coerulea*. *Marine Drugs* **16**, 341 (2018).

15 Espinosa, E. P., Koller, A. & Allam, B. Proteomic characterization of mucosal secretions in the eastern oyster, *Crassostrea virginica*. *Journal of proteomics* **132**, 63-76 (2016).

16 Ballard, K. R., Klein, A. H., Hayes, R. A., Wang, T. & Cummins, S. F. The protein and volatile components of trail mucus in the Common Garden Snail, *Cornu aspersum*. *PloS one* **16**, e0251565 (2021).

17 Perez-Riverol, Y. *et al.* The PRIDE database resources in 2022: a hub for mass spectrometry-based proteomics evidences. *Nucleic Acids Res* **50**, D543-d552, doi:10.1093/nar/gkab1038 (2022).
18 Johnson, M. *et al.* NCBI BLAST: a better web interface. *Nucleic acids research* **36**, W5-W9 (2008).
19 Thompson, J. D., Gibson, T. J. & Higgins, D. G. Multiple sequence alignment using ClustalW and ClustalX. *Current protocols in bioinformatics*, 2.3. 1-2.3. 22 (2003).
20 Letunic, I. & Bork, P. Interactive Tree Of Life (iTOL) v5: an online tool for phylogenetic tree display and annotation. *Nucleic acids research* **49**, W293-W296 (2021).
21 Potter, S. C. *et al.* HMMER web server: 2018 update. *Nucleic acids research* **46**, W200-W204 (2018).
22 Procter, J. B. *et al.* in *Multiple Sequence Alignment* 203-224 (Springer, 2021).
23 Shajahan, A., Supekar, N. T., Gleinich, A. S. & Azadi, P. Deducing the N- and O-glycosylation profile of the spike protein of novel coronavirus SARS-CoV-2. *Glycobiology* **30**, 981-988, doi:10.1093/glycob/cwaa042 (2020).
24 Bern, M., Kil, Y. J. & Becker, C. Byonic: Advanced Peptide and Protein Identification Software. *Current Protocols in Bioinformatics* **40**, 13.20.11-13.20.14, doi:https://doi.org/10.1002/0471250953.bi1320s40 (2012).
25 Kang, P., Mechref, Y., Kyselova, Z., Goetz, J. A. & Novotny, M. V. Comparative glycomic mapping through quantitative permethylation and stable-isotope labeling. *Analytical chemistry* **79**, 6064-6073 (2007).
26 Wu, Y. *et al.* N-Glycomic profiling reveals dysregulated glycans related to oral cancer using MALDI-MS. *Analytical and Bioanalytical Chemistry* **414**, 1881-1890 (2022).
27 Damerell, D. *et al.* in *Glycoinformatics* 3-15 (Springer, 2015).
28 Burgess, S. & Pinard, P. AZtec Wave–a New Way to Achieve Combined EDS and WDS Capability on SEM. *Microscopy and Microanalysis* **26**, 114-115 (2020).
29 Wu, G., Gotthardt, M. & Gollasch, M. Assessment of nanoindentation in stiffness measurement of soft biomaterials: kidney, liver, spleen and uterus. *Scientific reports* **10**, 1-11 (2020).
