# Supplementary material for: Comparative mucomic analysis of three functionally distinct Cornu aspersum Secretions
Source: Nat Commun. 2023 Sep 2;14:5361. doi: 10.1038/s41467-023-41094-z (PMC10475054; doi:10.1038/s41467-023-41094-z)
Supplement: Supplementary file 3 — Reporting summary [file 41467_2023_41094_MOESM3_ESM.pdf]

nature portfolio

Corresponding author(s): Adam B. Braunschweig

Last updated by author(s): Aug 1, 2023

Reporting Summary

Nature Portfolio wishes to improve the reproducibility of the work that we publish. This form provides structure for consistency and transparency in reporting. For further information on Nature Portfolio policies, see our Editorial Policies and the Editorial Policy Checklist.

Statistics

For all statistical analyses, confirm that the following items are present in the figure legend, table legend, main text, or Methods section.

n/a	Confirmed	
☐	☒	The exact sample size (*n*) for each experimental group/condition, given as a discrete number and unit of measurement
☐	☒	A statement on whether measurements were taken from distinct samples or whether the same sample was measured repeatedly
☒	☐	The statistical test(s) used AND whether they are one- or two-sided<br>*Only common tests should be described solely by name; describe more complex techniques in the Methods section.*
☒	☐	A description of all covariates tested
☒	☐	A description of any assumptions or corrections, such as tests of normality and adjustment for multiple comparisons
☐	☒	A full description of the statistical parameters including central tendency (e.g. means) or other basic estimates (e.g. regression coefficient) AND variation (e.g. standard deviation) or associated estimates of uncertainty (e.g. confidence intervals)
☒	☐	For null hypothesis testing, the test statistic (e.g. *F*, *t*, *r*) with confidence intervals, effect sizes, degrees of freedom and *P* value noted<br>*Give P values as exact values whenever suitable.*
☒	☐	For Bayesian analysis, information on the choice of priors and Markov chain Monte Carlo settings
☒	☐	For hierarchical and complex designs, identification of the appropriate level for tests and full reporting of outcomes
☒	☐	Estimates of effect sizes (e.g. Cohen's *d*, Pearson's *r*), indicating how they were calculated

Our web collection on statistics for biologists contains articles on many of the points above.

Software and code

Policy information about availability of computer code

Data collection	All software used in data collection are freely available and are as follows: FastQC v0.11.5, Trimmomatic v0.36, Trinity v2.4.0. No previously unreported software or code was used.
Data analysis	The following software used in data analysis are freely available and are as follows: Scaffold Q+ Scaffold_4.9.0, iTOL v5, Jalview v2.11.2.2, GlycoWorkbench 2.0, NanoScope Analysis 1.9. The following software were used with an institutional license: Byonic v5.0, SCIEX Data Explorer v2.2, Aztec v6.0. No previously unreported software or code was used.

For manuscripts utilizing custom algorithms or software that are central to the research but not yet described in published literature, software must be made available to editors and reviewers. We strongly encourage code deposition in a community repository (e.g. GitHub). See the Nature Portfolio guidelines for submitting code & software for further information.

Data

Policy information about availability of data

All manuscripts must include a data availability statement. This statement should provide the following information, where applicable:

- Accession codes, unique identifiers, or web links for publicly available datasets
- A description of any restrictions on data availability
- For clinical datasets or third party data, please ensure that the statement adheres to our policy

The mass spectrometry proteomics data have been deposited to the ProteomeXchange Consortium via the PRIDE partner repository with the dataset identifier

PXD035534 and 10.6019/PXD035534. RNA sequences that support the findings of this study have been deposited in Genbank with the primary BioSample accession codes SAMN29856567, SAMN29856568, SAMN29856569, SAMN29856570, SAMN29856571, SAMN29856572 and SRA accession codes SRR20337023, SRR20337022, SRR20337021, SRR20337019, SRR20337020, SRR20337018. Glycomics data and glycoproteomics data were submitted to GlycoPost database under the accession number GPST000297. The NCBI non-redundant protein database and PFAM protein database were used in this work. The authors declare that all other data that support the findings of this study are available within the paper and its supplementary information files.

Research involving human participants, their data, or biological material

Policy information about studies with human participants or human data. See also policy information about sex, gender (identity/presentation), and sexual orientation and race, ethnicity and racism.

Reporting on sex and gender	This research does not include human participants, their data, or biological material.
Reporting on race, ethnicity, or other socially relevant groupings	This research does not include human participants, their data, or biological material.
Population characteristics	This research does not include human participants, their data, or biological material.
Recruitment	This research does not include human participants, their data, or biological material.
Ethics oversight	This research does not include human participants, their data, or biological material.

Note that full information on the approval of the study protocol must also be provided in the manuscript.

Field-specific reporting

Please select the one below that is the best fit for your research. If you are not sure, read the appropriate sections before making your selection.

☒ Life sciences ☐ Behavioural & social sciences ☐ Ecological, evolutionary & environmental sciences

For a reference copy of the document with all sections, see nature.com/documents/nr-reporting-summary-flat.pdf

Life sciences study design

All studies must disclose on these points even when the disclosure is negative.

Sample size	No sample size calculation was performed. Several dozen visibly healthy and active animals were selected from a large pool for material collection. This sample size was chosen to provide enough secretory material for purification and analysis (0.1 - 10 mg of total purified, dried protein) without using washing methods that dilute the materials that are already > 90% water. This sample size also allowed for animals to be substituted when they appeared to be stressed.
Data exclusions	No data were excluded from the analyses.
Replication	Attempts to replicate purification results, glycomic analysis, elemental analysis, and mechanical property measurements were successful. Each of these studies were repeated independently three times. Sequencing analysis was conducted on six biological replicates to increase validity of the findings, and all six were successful.
Randomization	From the pool of animals provided, visibly healthy animals were selected and randomly distributed for material collection.
Blinding	Blinding was not possible for this study because visibly healthy animals were selected for material collection.

Reporting for specific materials, systems and methods

We require information from authors about some types of materials, experimental systems and methods used in many studies. Here, indicate whether each material, system or method listed is relevant to your study. If you are not sure if a list item applies to your research, read the appropriate section before selecting a response.

Materials & experimental systems

n/a	Involved in the study
☒	☐ Antibodies
☒	☐ Eukaryotic cell lines
☒	☐ Palaeontology and archaeology
☐	☒ Animals and other organisms
☒	☐ Clinical data
☒	☐ Dual use research of concern
☒	☐ Plants

Methods

n/a	Involved in the study
☒	☐ ChIP-seq
☒	☐ Flow cytometry
☒	☐ MRI-based neuroimaging

Animals and other research organisms

Policy information about studies involving animals; ARRIVE guidelines recommended for reporting animal research, and Sex and Gender in Research

Laboratory animals	This study did not include laboratory animals.
Wild animals	Cornu aspersum snails were provided by the Peconic Escargot (Cutchogue, NY) snail farm. Researchers traveled to the farm and handled all animals on-site. Snails selected were approximately one year old. Following biological material collection, animals were immediately returned to the farm unharmed. Five snails were sacrificed on-site via freezing in a dry ice-ethanol mixture for tissue preservation and collection.
Reporting on sex	Snails are hermaphrodites so animal sex has no bearing on this study.
Field-collected samples	Snails were cultured at room temperature and provided a diet of dirt, wild herbs, and cultivated herbs ad libitum. Physically active snails were washed with room temperature tap water to remove food and debris and placed into a plastic aquarium for mucus sample collection. Following material collection, snails were placed back into their original habitats.
Ethics oversight	Ethical approval was not required as Cornu aspersum snails are invertebrates.

Note that full information on the approval of the study protocol must also be provided in the manuscript.
